# Micro-computed tomography imaging reveals the development of a unique tooth mineralization pattern in mackerel sharks (Chondrichthyes; Lamniformes) in deep time

**DOI:** 10.1038/s41598-019-46081-3

**Published:** 2019-07-04

**Authors:** Patrick L. Jambura, René Kindlimann, Faviel López-Romero, Giuseppe Marramà, Cathrin Pfaff, Sebastian Stumpf, Julia Türtscher, Charlie J. Underwood, David J. Ward, Jürgen Kriwet

**Affiliations:** 1 University of Vienna, Department of Palaeontology, Vienna, Austria; 2 Haimuseum und Sammlung R. Kindlimann, Aathal-Seegräben, Switzerland; 3 Birkbeck, University of London, Department of Earth and Planetary Sciences, London, UK; 4 Natural History Museum, Department of Earth Sciences, London, UK

**Keywords:** Phylogenetics, Zoology, Taxonomy

## Abstract

The cartilaginous fishes (Chondrichthyes) have a rich fossil record which consists mostly of isolated teeth and, therefore, phylogenetic relationships of extinct taxa are mainly resolved based on dental characters. One character, the tooth histology, has been examined since the 19^th^ century, but its implications on the phylogeny of Chondrichthyes is still in debate. We used high resolution micro-CT images and tooth sections of 11 recent and seven extinct lamniform sharks to examine the tooth mineralization processes in this group. Our data showed similarities between lamniform sharks and other taxa (a dentinal core of osteodentine instead of a hollow pulp cavity), but also one feature that has not been known from any other elasmobranch fish: the absence of orthodentine. Our results suggest that this character resembles a synapomorphic condition for lamniform sharks, with the basking shark, *Cetorhinus maximus*, representing the only exception and reverted to the plesiomorphic tooth histotype. Additionally, †*Palaeocarcharias stromeri*, whose affiliation still is debated, shares the same tooth histology only known from lamniform sharks. This suggests that †*Palaeocarcharias stromeri* is member of the order Lamniformes, contradicting recent interpretations and thus, dating the origin of this group back at least into the Middle Jurassic.

## Introduction

Lamniform sharks include some of the most iconic shark species, like the great white shark (*Carcharodon carcharias*) and the biggest macropredatory shark that has ever roamed the world’s oceans, †*Otodus megalodon*^1–3^. Both, molecular and morphological data support the monophyly of this group, which today comprises seven families with 15 species. Together with the orders Carcharhiniformes, Orectolobiformes and Heterodontiformes they form the superorder Galeomorphii, which is the sister clade to the Squalomorphii (Hexanchiformes, Pristiophoriformes, Squatiniformes and Squaliformes)^4–8^. The oldest confirmed lamniform sharks are from the Valanginian (Early Cretaceous)^9^, but the origin of this group remains ambiguous, because the systematic position of †*Palaeocarcharias stromeri* from the early Tithonian (Late Jurassic) remains unclear as being either a stem lamniform^10,11^, an extinct sister group to lamniforms^12,13^, or sister to a clade comprising Carcharhiniformes and Lamniformes^14^.

A unique pattern of sharks is the tooth renewal with constantly forming series of teeth resulting in that functional teeth are shed and replaced in a constant and controlled succession (polyphyodont dentition)^15–17^. Teeth are initially formed within the dental lamina and during their development move from a lingual into a labial position in a conveyer belt like fashion^18,19^. The continuous shedding of teeth and the lack of a bony skeleton led to the preservation of a rich fossil record based predominantly on taxa known from isolated teeth only. Consequently, tooth characters such as crown and root morphologies or root vascularization patterns mostly are the only features that can be used to infer the systematic position of extinct sharks^16,17^.

Glickman^20,21^ attempted to resolve the systematic positions of fossil chondrichthyans based solely on tooth histologies of the crown and introduced the concept of histotype inferring. He distinguished between two different tooth histologies - the orthodont and the osteodont tooth histotypes. Accordingly, the orthodont type is characterized by the presence of a hollow pulp cavity which is encased by dentine that has tightly packed parallel tubules giving it a compact appearance (orthodentine)^22–24^. In contrast, teeth displaying the osteodont histotype have the pulp cavity filled by dentine that is composed of numerous vascular canals surrounded by concentric layers of dentine, similar to osteons in spongy bone (osteodentine)^22–24^, which intrudes from the root into the crown and fills the pulp cavity^25,26^.

Although the phylogeny of chondrichthyans has been drastically improved by adding more dental and morphological characters in recent years^5,27–29^, the tooth histotype concept still is used to distinguish elasmobranch groups, as in rajiform and myliobatiform batomorphs^17,30^ or in galeomorph sharks between lamniform and carcharhiniform sharks, with lamniforms displaying the osteodont tooth histotype and carcharhiniforms the orthodont histotype^26,31,32^, with one exception: the carcharhiniform shark *Hemipristis elongata* that was assumed to have the osteodont tooth histology^16,17,25,33^.

Recent examinations of the alleged osteodont carcharhiniform shark *Hemipristis*, however, revealed the presence of a third histotype - the pseudoosteodont tooth histotype^26^. Teeth of *Hemipristis* have an osteodentine core that fills the hollow pulp cavity and that is encased by a layer of orthodentine. This is in contrast to the tooth histology of recently examined lamniform sharks (Lamnidae and Alopiidae) which lack an orthodentine layer and only have osteodentine^26,31,34^. Recognition of the pseudoosteodont tooth histotype that is based on misinterpreted osteodont histologies makes a re-evaluation of previously interpreted osteodont histotypes in various elasmobranchs necessary to infer the taxonomic and systematic importance of tooth histotypes in sharks, rays, and skates.

Here we re-evaluate the tooth histotype of lamniform sharks based on teeth of eight fossil taxa, including the enigmatic galeomorph shark †*Palaeocarcharias stromeri* and 11 extant species using micro-computed tomography (micro-CT) and traditional tooth sections.

Previously published information about the tooth histology of other lamniform sharks that were not examined here was added to this study. This results in the description of tooth mineralization patterns of a wide range of species, from the basalmost lamniform sharks assigned to Eoptolamnidae (sensu Kriwet *et al*.^35^), or Pseudoscapanorhynchidae (sensu Herman^36^) to 14 of the 15 extant species (Supplementary Table S1). Therefore, this study represents the most comprehensive synopsis of tooth mineralization patterns in lamniform sharks to date and discusses the phylogenetic relevance of the tooth histotype for systematic interpretations and the origin of lamniform sharks in deep time.

## Results

### Tooth mineralization patterns in lamniform sharks

Micro-CT images of tooth files from the upper jaw (palatoquadrate cartilage, PC) of the basking shark (*Cetorhinus maximus*) (7-692/RZ) and the left upper jaw (LPC) of the crocodile shark (*Pseudocarcharias kamoharai*) (7-693/RZ) were 3D reconstructed and virtually sectioned to examine the tooth mineralization sequence of both species. The tooth mineralization sequences were consistent through all tooth files of each investigated species, with little variations due to different numbers of teeth per tooth file. The code specification of the abbreviations used here is depicted in the Material and Methods section.

**Figure 1:**
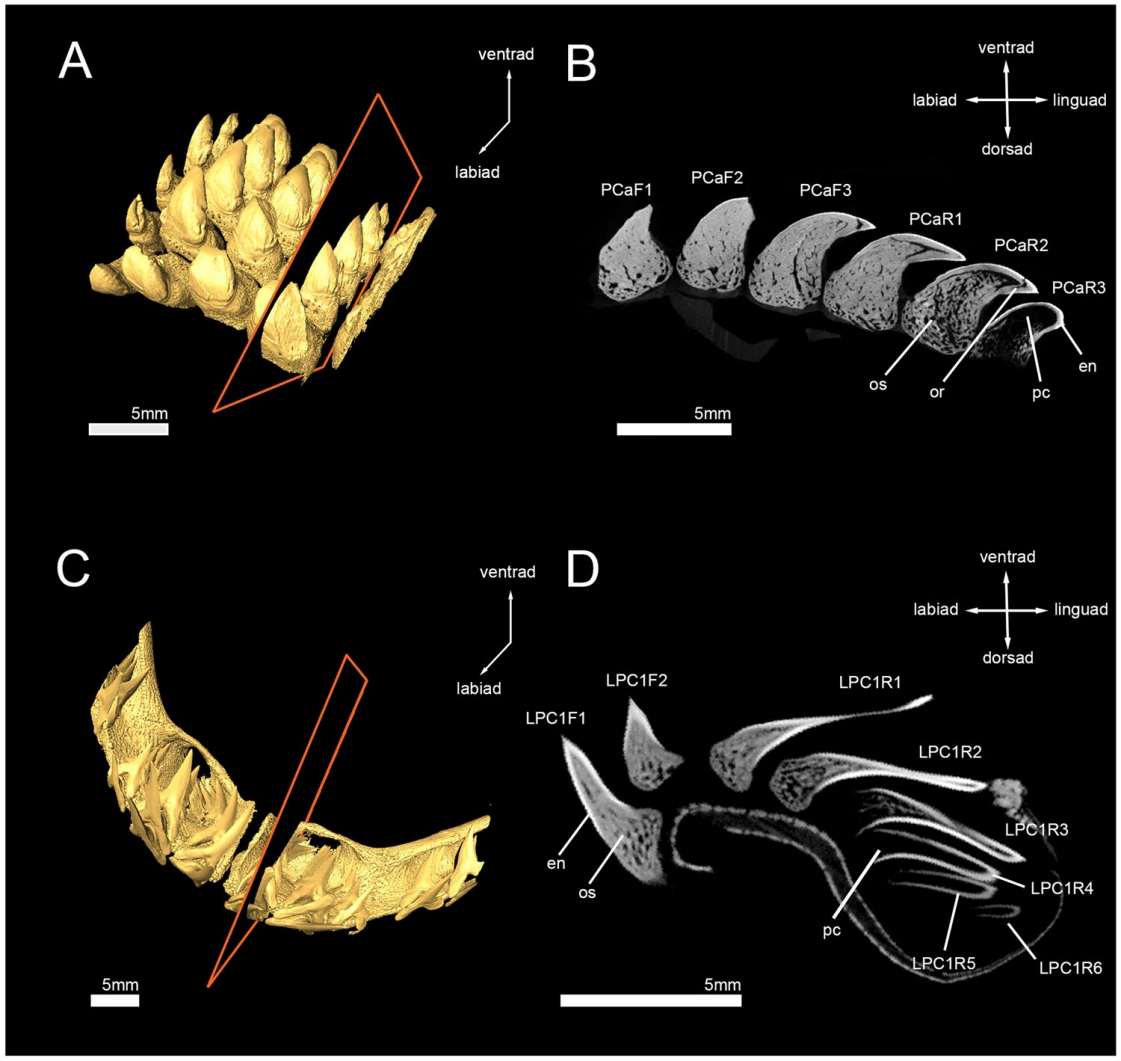
3D micro-CT isosurfaces of the upper jaws and 2-D images of the virtually sectioned tooth row reveal two different patterns of tooth mineralization in lamniform sharks. The basking shark *Cetorhinus maximus* (7-692/RZ) (**A**,**B**) develops two layers of dentine (orthodentine and osteodentine representing the pseudoosteodont histotype), while the crocodile shark *Pseudocarcharias kamoharai* (7-693/RZ) (**C**,**D**) lacks orthodentine and only develops osteodentine (osteodont histotype). en, enameloid; or, orthodentine; os, osteodentine; pc, pulp cavity.

**Figure 2:**
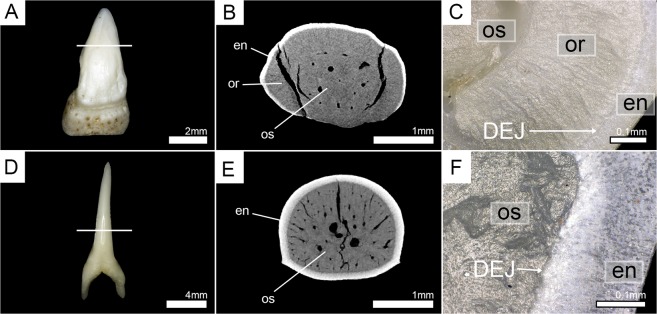
Horizontal virtual micro-CT sections and manual tooth sections of the basking shark (*Cetorhinus maximus*, Cetorhinidae) (EMRG-Chond-T-24) and the crocodile shark (*Pseudocarcharias kamoharai*, Pseudocarchariidae) (EMRG-Chond-T-28). (**A**) picture of a functional tooth prior to the tooth section, (**B**) virtual section, and (**C**) tooth section of *C*. *maximus* under normal light illustrating the presence of two layers of dentine - compact orthodentine surrounding a core of osteodentine. (**D**) picture of a functional tooth prior to the tooth section, (**E**) virtual section, and (**F**) tooth section of *P*. *kamoharai* under polarized light illustrating the presence of one layer of dentine - osteodentine. White lines indicate the approximate plane of the sections. do, dentinal osteons; en, enameloid; or, orthodentine; os, osteodentine.

*Pseudocarcharias kamoharai* has five to eight teeth in tooth files of the upper jaw, with zero to two functional teeth per tooth file. In the youngest developmental stages, the only mineralized structure of the tooth is the superficial enameloid. The enameloid first starts to mineralize at the apex (LPC1R6) and mineralization continues to the tooth crown base (LPC1R5). Mineralization of the enameloid is completed in LPC1R4. Until this position, the osteodentine formation has neither started in the root, nor in the crown and enameloid is the only mineralized structure. Osteodentine starts forming in the root and in the center of the crown simultaneously after completion of enameloid (LPC1R3), until it has fully filled the pulp cavity (LPC1R1 & LPC2R2). LPC1R1 is already fully mineralized, but not in a functional position and, therefore, regarded as the first replacement tooth. LPC1F1 and LPC1F2 are fully mineralized and are in an erect position on the outer edge of the jaw cartilage, allowing them to be utilized. During tooth development, no orthodentine can be identified at any stage, resulting in fully mineralized teeth consisting of only one sort of dentine – osteodentine (Fig. 1C,D). The virtual section of an isolated functional tooth also demonstrates the presence of only one layer of dentine, which is traversed by small canals and surrounded only by the hypermineralized enameloid (Fig. 2E). A manual tooth section confirms this, showing that dentinal osteons are spreading throughout the whole crown and are also present in close proximity or next to the enameloid (Fig. 2F). The presence of osteodentine and the absence of orthodentine implies the osteodont tooth mineralization pattern for teeth of *P*. *kamoharai* (Figs 1 and 2; Supplementary Tables S2 and S3).

The basking shark, *Cetorhinus maximus* has five to seven teeth within tooth files of the fractional part of the upper jaw of which three to four can be regarded as functional teeth because of their position along the margin of the jaw cartilage. Teeth have already an enameloid cap covering the whole of the crown in the earliest mineralization stages. A thin layer of orthodentine is present and osteodentine in the root already has started to form at this stage (PCaR3). In the adjacent file, only enameloid is present at the corresponding position, but no dentine (PCbR3) (Supplementary Fig. S1). Orthodentine can be distinguished from enameloid due to different densities. Dense tissues are represented by lighter shades (the hypermineralized enameloid is typically white in our reconstructions) and less dense tissues by darker shades (dentine is typically grey in our reconstructions). Osteodentine starts forming in the root and basally in the crown along the walls underneath the orthodentine. A hollow pulp cavity remains in the center of the tooth (PCaR3). During the next stages, the orthodentine layer becomes thicker and is fully mineralized in the first replacement tooth PCaR1. After the formation of osteodentine in the earliest stages, it intrudes basally into the pulp cavity until it fully fills the center of the crown. The osteodentine in the root is fully mineralized at the same developmental stage as orthodentine (PCaR1), but not in the crown until the next stage (PCaF3). The first replacement tooth (PCaR1) is already in a functional position, but the osteodentine in the crown has not fully filled the pulp cavity at this point. Therefore, completion of the mineralization process in the crown determines the earliest functional tooth (PCaF3) (Fig. 1A,B; Supplementary Tables S2 and S4). Manual and virtual tooth sections of an isolated functional tooth confirm the presence of both types of dentine - a dentine core which is made up of both, dentinal osteons and interosteonal tissue, giving it a spongiose appearance (osteodentine), which is surrounded by a prominent layer of dentine, lacking any vertical canals or pores but horizontal tubules, which are arranged parallel to each other and give the dentine layer a very compact appearance (orthodentine). The development of both, orthodentine and osteodentine in the crown shows that *C*. *maximus* has a pseudoosteodont mineralization pattern (Figs 1 and 2; Supplementary Tables S2 and S4).

**Figure 3:**
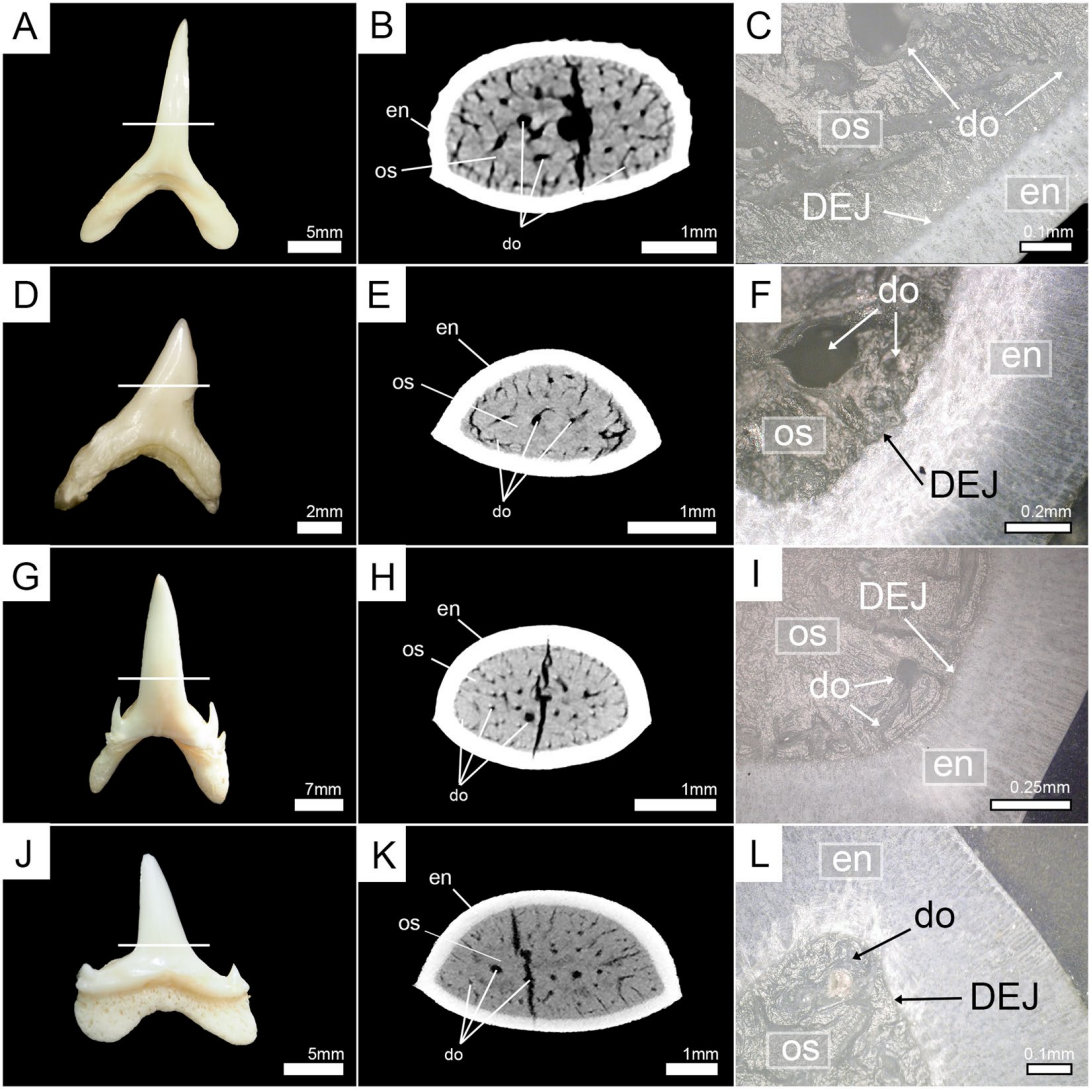
Horizontal virtual micro-CT sections and manual tooth sections of extant lamniform sharks under normal light. (**A**–**C**) goblin shark (*Mitsukurina owstoni*, Mitsukurinidae) (EMRG-Chond-T-1); (**D**–**F**) common thresher (*Alopias vulpinus*, Alopiidae) (EMRG-Chond-T-27); (**G**–**I**) smalltooth sand tiger (*Odontaspis ferox*, Odontaspididae) (EMRG-Chond-T-2); (**J**–**L**) porbeagle shark (*Lamna nasus*, Lamnidae) (EMRG-Chond-T-4). White lines indicate the approximate plane of the sections. do, dentinal osteons; en, enameloid; os, osteodentine.

**Figure 4:**
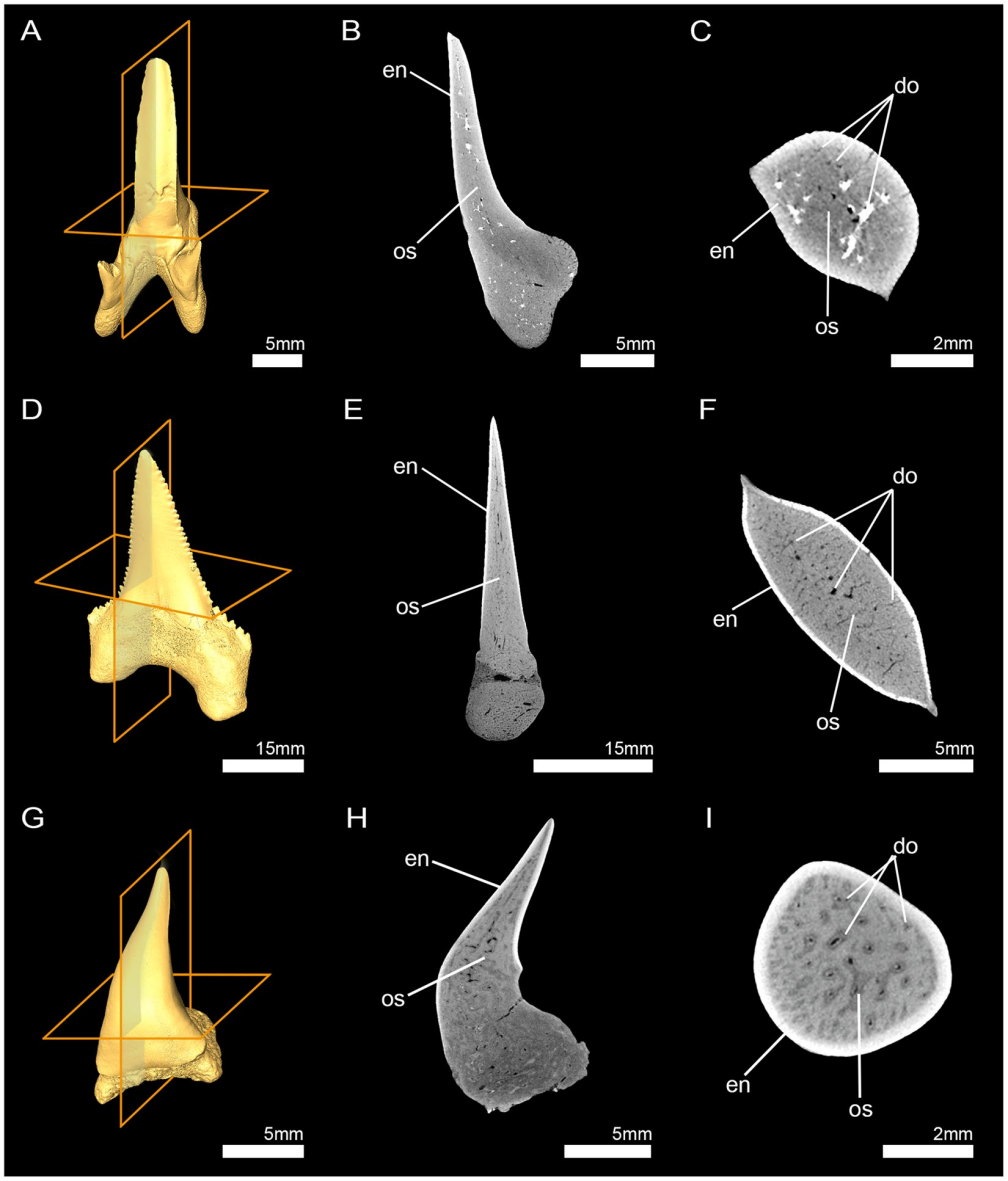
3D reconstructions and virtual micro-CT sections (sagittal and axial plane) of fossil lamniform shark teeth. (**A**–**C**) †*Leptostyrax* sp. (†Eoptolamnidae (sensu Kriwet *et al*.^35^)) (Inv.nr. 7–690); (**D**–**F**) †*Palaeocarcharodon orientalis* (Lamniformes incertae fam.) (EMRG-Chond-T-50); (**G**–**I**) *Megachasma pelagios* (Megachasmidae) (EMRG-Chond-T-44). do, dentinal osteons; en, enameloid; os, osteodentine.

To clarify which of the two mineralization patterns is present among the remaining members of the order Lamniformes, teeth of nine additional extant species were micro-CT scanned and virtually sectioned to determine their tooth histology. Additionally, teeth of all extant specimens (except the megamouth shark, *Megachasma pelagios*) also were manually sectioned horizontally and compared to the high-resolution images of the virtually sectioned teeth. Both methods demonstrate the presence of only one layer of dentine - osteodentine. Therefore, all extant species (except for *Cetorhinus maximus*) display the osteodont tooth histotype (Figs 2–4, Supplementary Fig. S2).

### Tooth histology patterns in fossil lamniform sharks

To clarify the plesiomorphic condition for lamniform sharks, fossil teeth of seven selected taxa (including supposedly basal (i.e. Eoptolamnidae (sensu Kriwet *et al*.^35^) and derived taxa) of extinct lamniform sharks were examined using micro-computed tomography images. Additionally, an isolated tooth of the extant species *Megachasma pelagios* from the Pliocene (2.5–5 mya) is included here as well.

Rendered high resolution micro-CT images of the virtual sections (in axial and sagittal view) reveal the absence of orthodentine in teeth of †*Leptostyrax* sp. (†Eoptolamnidae (sensu Kriwet *et al*.^35^)), †*Palaeocarcharodon orientalis* (Lamniformes incertae fam.), and *Megachasma pelagios* (Megachasmidae). The hypermineralized enameloid (white) is clearly distinguishable from the dentine (grey) as a result of density differences. From the core of the tooth to the enameloid-dentine border, the entire dentine layer is traversed by dentinal osteons (Fig. 4). The same histology pattern can be identified in teeth of †*Scapanorhynchus rapax* (Mitsukurinidae) and †*Squalicorax pristodontus* (†Anacoracidae), which thus also exhibit the osteodont tooth histotype (Supplementary Fig. S3).

**Figure 5:**
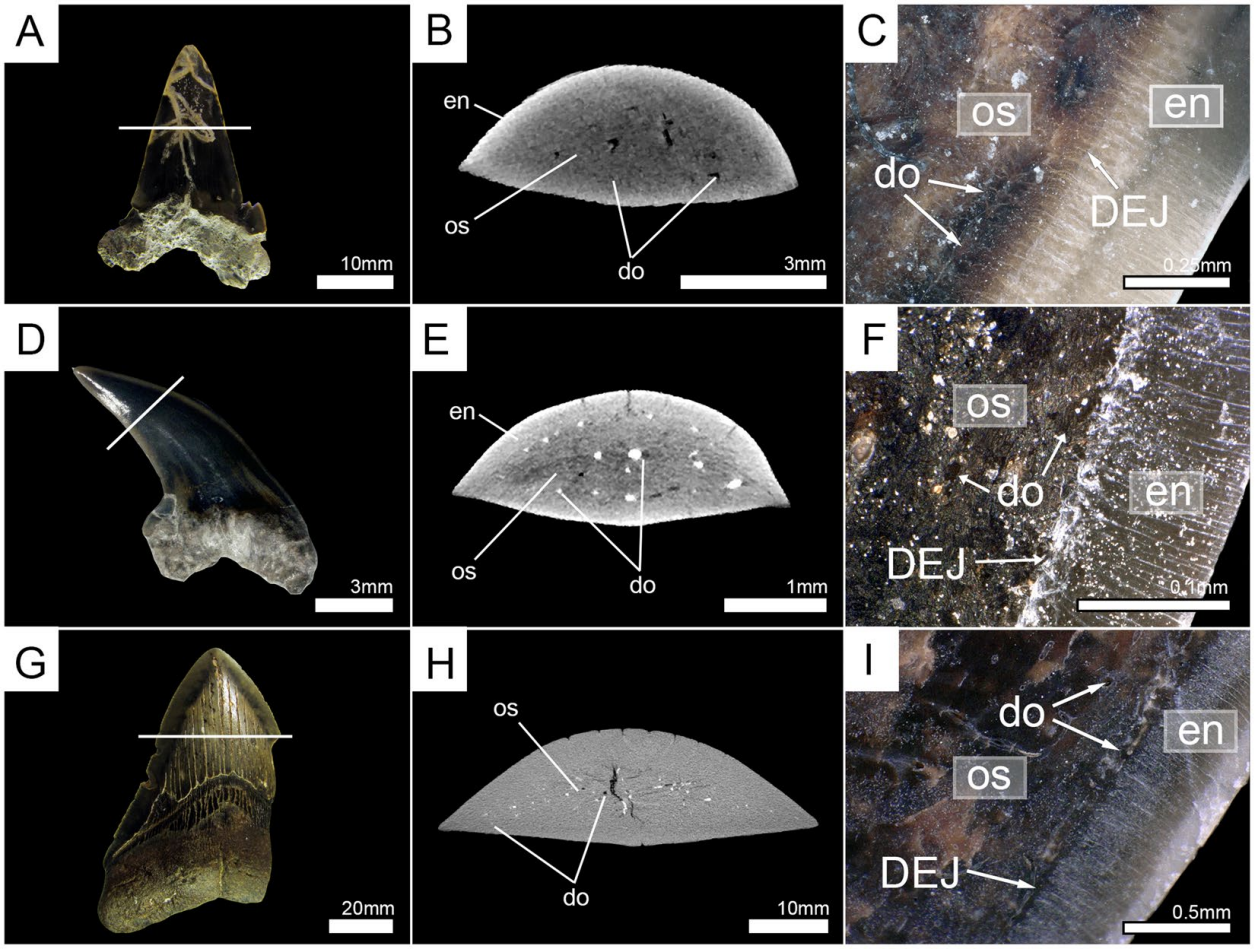
Horizontal virtual micro-CT sections and manual tooth sections of extinct lamniform sharks. (**A**–**C**) †*Dwardius woodwardi* (Lamniformes incertae fam.) (EMRG-Chond-T-53); (**D**–**F**) †*Squalicorax* sp. (†Anacoracidae) (EMRG-Chond-T-54); (**G**–**I**) †*Otodus megalodon* (†Otodontidae) (EMRG-Chond-T-57). White lines indicate the plane of the sections. do, dentinal osteons; en, enameloid; os, osteodentine.

In three cases (teeth of †*Dwardius woodwardi* (Lamniformes incertae fam.), †*Squalicorax* sp. (†Anacoracidae), and †*Otodus megalodon* (†Otodontidae)) the presence or absence of orthodentine could not be properly identified based solely on micro-CT images. The enameloid-dentine border was less well defined in micro-CT images than in other examined teeth and some areas of dentine were indistinct, probably due to taphonomic processes, not allowing to identify the presence or absence of dentinal osteons (Fig. 5B,E,H). This was especially evident in †*Otodus megalodon*, where tooth enameloid could not be distinguished from the dentinal core and only a few coarse canals were visible (Fig. 5H). Therefore, the tooth crowns were manually sectioned horizontally and examined under a light microscope. These tooth sections demonstrate the presence of dentinal osteons, traversing the entire dentine core from the center to the enameloid-dentine border (Fig. 5C,F,I). In †*Dwardius woodwardi* small dentinal tubuli are visible that originate in the osteons and penetrate the adjacent enameloid. Since they originate in the osteons they should be regarded as extensions of the osteon rather than representing an additional dentine layer. Orthodentine was not identified in any fossil tooth and all examined fossil taxa therefore have the osteodont tooth histotype.

### Tooth histology of †*Palaeocarcharias stromeri*

**Figure 6:**
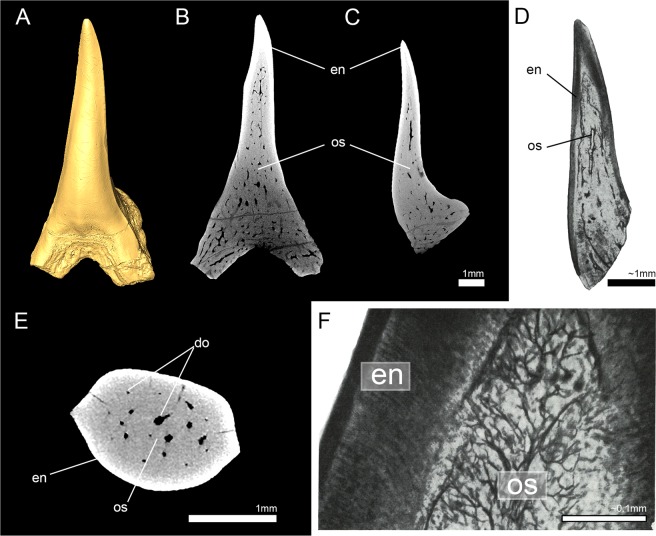
High resolution micro-CT images of virtual tooth sections and manual tooth sections of the original description of †*Palaeocarcharias stromeri*. (**A**) 3D reconstruction, (**B**,**C**,**E**) virtual tooth sections in (**B**) frontal, (**C**) sagittal and (**E**) axial view. (**D**,**F**) are modified pictures of the manual tooth sections from the original description of †*P*. *stromeri* by Beaumont^10^. Do, dentinal osteons; en, enameloid; os, osteodentine.

The systematic position of †*Palaeocarcharias stromeri* still has to be considered as ambiguous despite all recent advances (see supplementary information (Supplementary Discussion 1) for a discussion of the most recent phylogenetic analysis^14^). Here, tooth histology can provide additional and important information. For this, a tooth of the holotype (JME-SOS-2294) was scanned using micro-computed tomography. Virtual sections through three planes (frontal, sagittal, and axial) show a dentine core that is entirely traversed by a network of dentinal osteons (osteodentine) and covered by enameloid. The dentinal osteons are distributed within the entire dentine layer, from the center of the crown to close to the enameloid-dentine border. A compact layer of dentine (orthodentine) between the osteodont core and the hypermineralized enameloid is not present in the type specimen of †*Palaeocarcharias stromeri*, which therefore represents the osteodont histotype (Fig. 6B,C,E).

## Discussion

### Tooth mineralization patterns in sharks of the order Lamniformes

We identified two different tooth mineralization patterns within lamniform sharks resulting in the osteodont and pseudoosteodont histotypes. According to our results, the basking shark (*Cetorhinus maximus*) is the only lamniform shark having the pseudoosteodont tooth histotype, consisting of an osteodentine core that is covered by orthodentine. All other lamniform sharks displayed the osteodont tooth histotype, developing only one type of dentine (osteodentine). There was no lamniform species that showed the orthodont tooth histotype (a hollow pulp cavity surrounded by orthodentine) that is known from carcharhiniform sharks^25,26,31–33,37–41^. Teeth of the great white shark (*Carcharodon carcharias*)^31,32,42,43^, shortfin mako (*Isurus oxyrinchus*)^26,34^, and the salmon shark (*Lamna ditropis*)^44^ are also lacking orthodentine and thus 13 out of 14 examined extant lamniform shark species follow an osteodont tooth mineralization pattern. Unfortunately, there is no data for the longfin mako (*Isurus paucus*), but as all lamnids as well as its closest relative, *Isurus oxyrinchus*, have osteodont teeth^26,34^, it seems eligible to assume that its teeth also follow an osteodont mineralization pattern. Additionally, teeth of extinct species, from the assumed basal-most family †Eoptolamnidae (sensu Kriwet *et al*.^35^) (†*Leptostyrax* sp.) from the Albian/Cenomanian (Early Cretaceous, 94–113 mya) to †*Otodus megalodon* from the Miocene (Neogene, 5–23 mya) all showed the osteodont histotype. The latter species was previously described as being osteodont^45^, but also pseudoosteodont^46^. In both studies appropriate tooth sections were not prepared but fractured parts of the tooth were only superficially inspected. The tooth section prepared for this study unambiguously demonstrates that the whole interior of the crown is filled with osteodentine, while orthodentine is absent supporting †*O*. *megalodon* to be osteodont. The osteodont tooth histology for lamniforms is also confirmed by a number of publications: for a more detailed list, see Supplementary Table S1.

### Phylogenetic implications of different tooth histotypes and the origin of osteodonty

Many sharks, rays and skates (Elasmobranchii) previously have been considered to have the osteodont histotype according to the traditional definition of histotypes (pulp cavity filled with osteodentine)^20,21,25^: Myliobatiformes^22^, *Ptychodus*^22,47,48^, Hexanchiformes^41^, Squatiniformes^49^, Heterodontiformes^22^, Orectolobiformes^49^, *Hemipristis* spp. (Carcharhiniformes)^25,26,40^, Lamniformes^26,31,34,43^. However, tooth crowns of these groups consist of both, ortho- and osteodentine and, therefore, should be referred to as being pseudoosteodont sensu Jambura *et al*.^26^. Consequently, Lamniformes (except for *Cetorhinus*) is the only group in which osteodentine alone constitutes the interior of the tooth crown and, therefore, should be the only group to be referred to as being osteodont.

**Figure 7:**
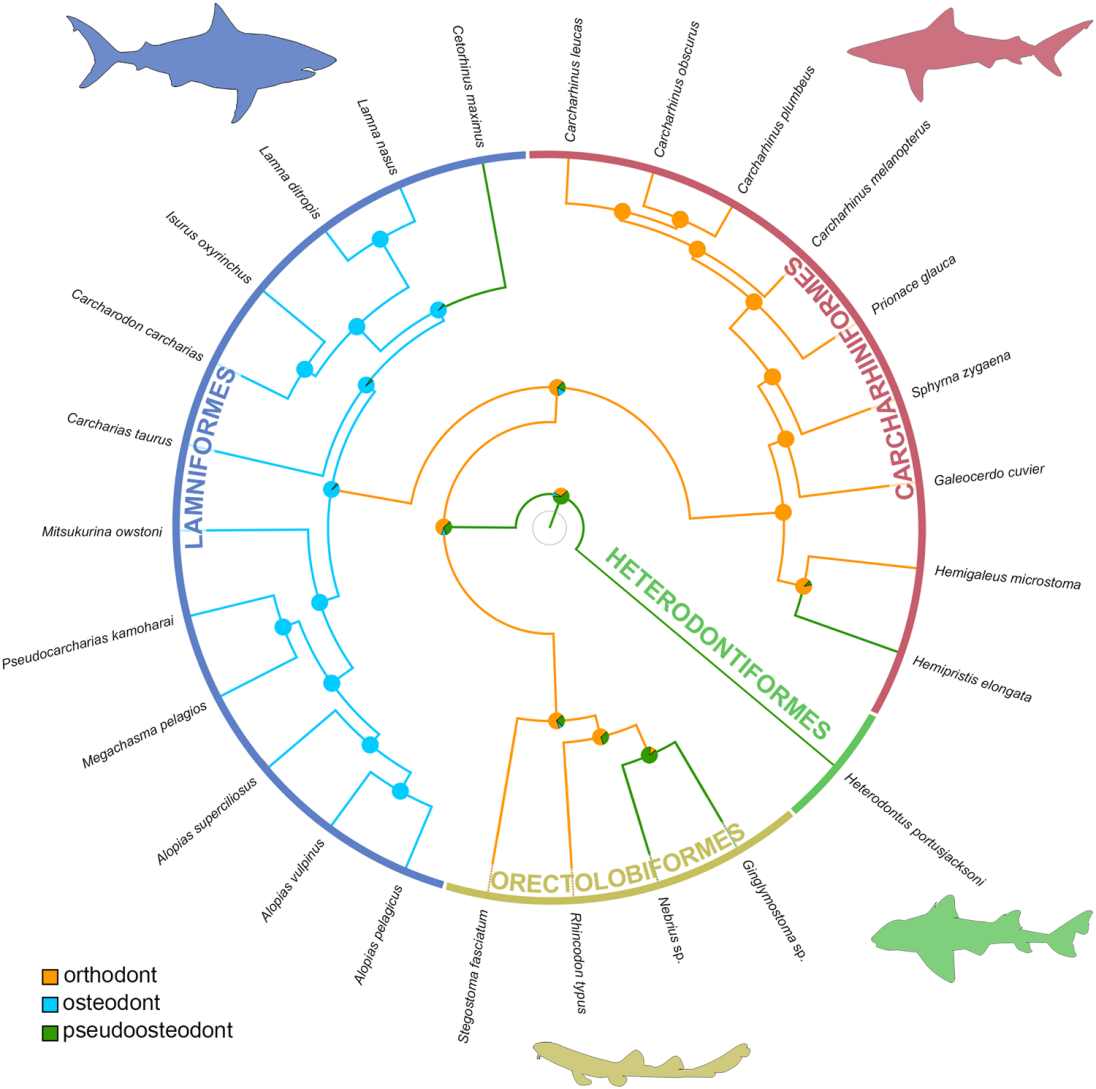
Stochastic character mapping of tooth histotypes and ancestral state reconstructions in sharks of the superorder Galeomorphii. The maximum credibility tree of the galeomorph sharks is based on whole mtDNA sequences. Ancestral states at the nodes are coded as pie charts proportions of the probability distribution, calculated from 100 stochastic mappings for the three histotypes present (orthodonty, osteodonty and pseudoosteodonty).

Many Palaeozoic and hybodontiform sharks (the putative sister group to modern sharks^50,51^) are also referred to as being osteodont^22,52–55^ according to the traditional definition of histotypes. However, as in modern groups (except for the lamniform sharks) both, orthodentine and osteodentine form the interior of the crown and, therefore, they have the pseudoosteodont histotype. There seems to be only one exception: apparently the teeth of †*Aztecodus harmsenae*, a Devonian chondrichthyan, also lack orthodentine^53^. The presence of pseudoosteodonty in Palaeozoic sharks, †Hybodontiformes and in many extant elasmobranchs (sharks, rays and skates) strongly indicates that this is the plesiomorphic condition for the modern sharks, and not a modification of the orthodont tooth histotype as previously suggested^26,40^. The osteodont tooth histotype exclusively found in sharks of the order Lamniformes represents a highly derived synapomorphic condition for this group within the elasmobranch fishes (Fig. 7).

The basking shark *Cetorhinus maximus* however, represents a deviation from the general lamniform pattern in that it displays the pseudoosteodont tooth histology. In phylogenetic analyses based solely on tooth morphology, *C*. *maximus and Megachasma pelagios* formed a clade at the base of the lamniform sharks, representing a primitive sister group to other lamnid sharks^56^. Although this would explain a plesiomorphic state of the tooth histology in *Cetorhinus* to some extent, it conflicts the derived state in its putative sister group *Megachasma*. Furthermore, other morphological characters and molecular data suggest *C*. *maximus* to be a highly derived lamniform shark^4–6,8^, indicating that teeth of both planktivores became vestigial and, therefore, dental characters were reduced or lost completely^56,57^. This might also be the case for the tooth histology of *C*. *maximus* which reverted to a plesiomorphic state. However, *M*. *pelagios*, the second planktivorous species of this order still shares its derived tooth histology with all other lamniform sharks and, therefore, does not support an ecophenotypic link between tooth histology and feeding behaviour. What is also curious about the tooth histology in *Cetorhinus maximus* is that it was not reverted to the most probable ancestral state of lamniform and carcharhiniform sharks (orthodont), but to the ancestral state of all galeomorph sharks (pseudoosteodont) (Fig. 7). The reason for this reversal of the tooth mineralization pattern in *C*. *maximus* remains ambiguous, but seemingly is neither linked to its phylogenetic position, nor to its planktivorous feeding behaviour.

### †*Palaeocarcharias stromeri* and the origin of lamniform sharks

High resolution micro-CT images of a tooth of the holotype specimen of †*Palaeocarcharias stromeri* (JME-SOS-2294) display two types of tissues - a prominent outer layer of hypermineralized tissue (enameloid) and a core of less dense tissue (dentine). Dentinal osteons traversed the whole dentine core and were also present very close to the enameloid layer, which is characteristic for osteodentine. There were no signs of a compact orthodentine layer which can be found in pseudoosteodont teeth, thus teeth of †*P*. *stromeri* consist of only one layer of dentine (osteodentine) and display the osteodont histotype, which is characteristic for lamniform sharks^26,31,34,41^.

Our findings contradict the original description of the tooth histology of †*P*. *stromeri* by Beaumont^10^, who indicated the presence of three layers of dentine - a core of “trabecular dentine” (osteodentine) that was surrounded by a mantle of orthodentine, which again was covered by vitrodentine^10^ (vitrodentine is one of many synonyms that were used for enameloid before its true nature was resolved^58^). The presence of both, orthodentine and osteodentine, within the crown would suggest that †*P. stromeri* displays the pseudoosteodont histotype. According to the most recent study on †*P*. *stromeri*, vitrodentine and orthodentine were misinterpreted in the original work and instead are components of the multi layered enameloid^14^. Vitrodentine resembles the SCE (‘Single Crystallite Enameloid’) unit, while the tissue described as orthodentine in fact was the BCE (‘Bundled Crystallite Enameloid’) unit of the enameloid. Misinterpretations of the BCE unit (or parts of it) to be orthodentine occurred also in other studies, i.e. for *Lamna* sp.^59^ and *Lamna nasus*^60^. Berkovitz and Shellis^61^ reported the presence of orthodentine in *Carcharias* sp. However, dentinal tubules in the tissue they identified as orthodentine in their figure (figure 11.30)^61^ do not originate in this tissue but come from the adjacent osteodentine which is known to occur at the dentine-enameloid junction^31^. Furthermore, the presence of a sharp junction between osteodentine and “orthodentine”, which is known from the enameloid-dentine border, but not from dentine-dentine borders^31,61^ is another indicator for this misinterpretation. Therefore, it is apparent that *Carcharias* sp. has the osteodont tooth histotype reported here for *Carcharias taurus* and other lamniform sharks.

Another indicator for the multi-layered enameloid being misinterpreted as orthodentine in the original work of †*P*. *stromeri* is given by our micro-CT images. Computed tomography discriminates between tissues of different densities - in our case, the very dense hypermineralized enameloid appears white, while the less dense dentine is grey. If we compare the virtual section in sagittal view (Fig. 6C) with the original tooth section (also in sagittal view) (Fig. 6D), we can see that the hypermineralized tissue in the virtual section (white) resembles the combined layers of “vitrodentine” and “orthodentine” in the original tooth section (dark) in thickness. If only the thin layer of “vitrodentine” in the original tooth section was enameloid, the hypermineralized structure in the micro-CT scan would be much thinner and less prominent, since the enameloid has a higher mineral content and, thus, density than dentine^58,62,63^, which is also visible on the micro-CT images^26,34^. Therefore, the micro-CT images prove the misinterpretation of orthodentine in †*P*. *stromeri*, which means that it does not display the pseudoosteodont tooth histotype, but the osteodont histotype, which only occurs in lamniform sharks.

Although †*P*. *stromeri* is known from well-preserved articulated material, only a few studies were conducted to resolve the systematic position of this species, which was in debate for decades^12,13^. In the original description, †*P*. *stromeri* was placed at the base of the Lamniformes^10^. Other authors agreed that the tooth morphology is characteristic for lamniform sharks^11,16,17^ but the body form was similar to that of orectolobiform sharks^11^, making it a transitional taxon between both clades or the basal sister group of lamniforms^12,13^. In the most recent work on the phylogenetic position of †*P*. *stromeri*, it was suggested to be the sister to the clade Carcharhiniformes + Lamniformes^14^. Unfortunately, this phylogenetic hypothesis is inconclusive (Supplementary Discussion S1). Consequently, it is most parsimonious to assume that the osteodont tooth histotype is a unique feature for Lamniformes and thus we conclude that the osteodont tooth histology of †*P*. *stromeri* adds very strong support of this shark being the oldest known lamniform shark. Therefore, the origin of this group dates back at least into the Middle Jurassic (Bathonian)^64^, a period when major diversifications of elasmobranch fishes took place^65,66^.

### Strengths and weaknesses of micro-CT imaging

Similar to previous studies, micro-CT imaging turned out to be a powerful tool for non-invasive investigations of internal structures^26,31,43,67^. This is especially evident in examining tooth mineralization processes within tooth files in extant lamniform sharks. However, our study also points out weaknesses of micro-CT imaging, especially for fossil specimens related either to taphonomic alterations or to insufficient resolution of the CT scan. This is especially apparent in the tooth of †*Otodus megalodon*, in which the enameloid was not distinguishable from the dentine and the peripheral vascular system with its tiny canaliculi was not detectable in the micro-CT images, but was visible in manual tooth section. The latter effect is easily explained by the low resolution (around 30 µm), which was caused by the size of the tooth. In extant sharks, enameloid has a much higher degree of crystallinity and a very low organic content compared to dentine^62,63^, which makes it appear denser in micro-CT images^26,34^. The poor results for fossil teeth can be distorted by diagenetic processes leading to changes in the chemical constitution of enameloid^63,68^ resulting in less differences of densities between enameloid and dentine. Nonetheless, in most cases micro-CT scanning generated images that sufficiently resolved the internal structures of both, extant and fossil shark teeth without damaging or destroying the material and therefore, should be regarded as a reliable non-invasive alternative to conventional thin sectioning.

## Material and Methods

### Material

Teeth and jaws of 11 extant and seven extinct taxa of sharks of the order Lamniformes were examined. Additionally, a tooth of the holotype specimen of †*Palaeocarcharias stromeri* (JME-SOS-2294) housed in the Jura Museum Eichstätt, Germany, the putative basal most lamniform shark from the Upper Jurassic (Tithonian), is included in this study.

Extant material consisted of two jaws (basking shark *Cetorhinus maximus* (Inv.nr. 7-692/RZ), crocodile shark *Pseudocarcharias kamoharai* (Inv.nr. 7-693/RZ)) and isolated teeth of all 11 species. The fossil material consisted exclusively of isolated teeth and includes species from the Mesozoic and Cenozoic Era (five and three species respectively). Daggers preceding taxon names denote extinct species (Supplementary Table S5).

### Tooth terminology

**Figure 8:**
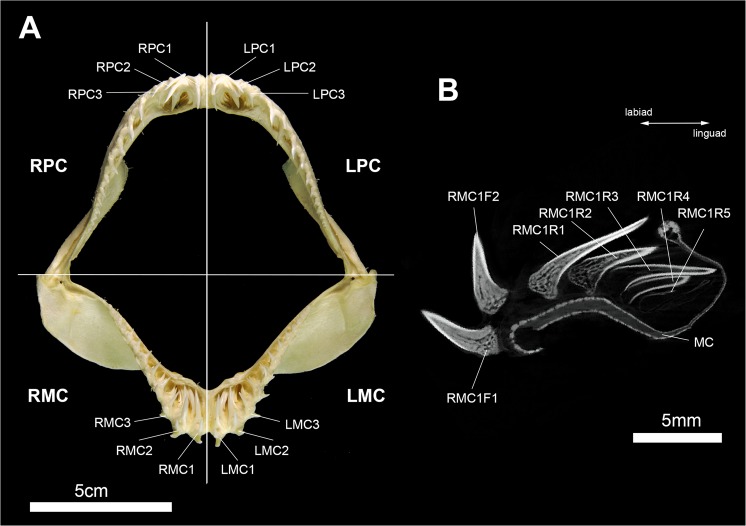
Terminology used to describe the topology of teeth within the jaws and tooth files. (**A**) Jaws of *Pseudocarcharias kamoharai* (Inv.nr. 7-693/RZ) in frontal view, (**B**) virtual section through the tooth file RMC1. F, functional tooth; F1, first (oldest) functional tooth; F2, second functional tooth; LMC, left Meckel’s cartilage; LPC, left palatoquadrate cartilage; MC, Meckel’s cartilage (lower jaw); PQ, palatoquadrate cartilage (upper jaw); R, replacement tooth; R1, first (oldest) replacement tooth; R2, second replacement tooth; R3, third replacement tooth; R4, fourth replacement tooth; R5, fifth replacement tooth; RMC, right Meckel’s cartilage; RPC, right palatoquadrate cartilage.

To specify the tooth position within the jaw, we employed a previously published code^26,34^. The first four letters define the position of the tooth file (the developmental sequence of replacement and functional teeth sensu Moyer *et al*.^31^), if it is right (R) or left (L) of the symphysis or coming from the upper (palatoquadrate PC) or lower jaw (Meckel’s cartilage MC). The number following the first three letters determines the position of the file distally to the symphysis. For example, the tooth file illustrated in Fig. 1B is the first file right to the symphysis in the lower jaw and, therefore, coded as RMC1. We distinguished between functional (F) and replacement teeth (R), with functional teeth being fully mineralized and in an erect or semi-erect position, allowing them to be utilized for food gathering (e.g. cutting, grasping, etc.). Replacement teeth are located lingually to the functional teeth and are not fully mineralized at this point of development. The tooth position within the tooth file is numbered, thus the first functional tooth of the RMC1 file is RMC1F1, the first replacement tooth of the same file is RMC1R1 (Fig. 8).

### Micro-CT scanning and imaging

Tooth mineralization and histology patterns for each species were investigated using a SkyScan1173 micro-CT device (Bruker/Skyscan, Kontich, Belgium) at the Department of Palaeontology (University of Vienna, Austria). Settings for each specimen are provided in the Supporting Information section (Supplementary Table S6). The generated slice file stacks were loaded into the software package DataViewer (version 1.5.1.2 (64 bit), SkyScan (Bruker micro-CT, Kontich, Belgium)) and Amira software package (version 5.4.5, FEI Visualization Sciences Group, Oregon, USA) to visualize and investigate 2D and 3D images of the studied material. This allowed us to set clipping planes through the jaws and teeth with different angles and digitally dissect through the material to examine the internal anatomy. Editing colour balance, contrast and labeling of the resulting 2D images was conducted in Adobe Photoshop CS6 (version 13.0, Adobe Systems, San José, USA).

### Tooth sectioning

The quality of the results was tested by comparing the digital sections with actual tooth sections. For this, tooth sections were prepared for all extant specimens and three fossil specimens (†*Dwardius woodwardi*, †*Otodus megalodon*, †*Squalicorax* sp.). For better handling of the small teeth, they were embedded in an adhesive medium using the two-component adhesive Araldite 2020/A and Araldite 2020/B, which were merged with a ratio of 100:30. The embedded teeth were cut horizontally through the crown and the exposed surface was polished using grinding powder (grain size 600 and 1000). Afterwards, the surface was treated with a 2 molar HCl solution for 10–60 sec and examined under the digital microscope Keyence VHX-6000 (Keyence International, Belgium). Pictures of the teeth were taken prior the sectioning process with an Olympus-OMD E5 mirrorless camera or the digital microscope.

### Phylogenetic tree and ancestral state reconstruction

A phylogeny for 26 galeomorph sharks with known tooth histology was built from whole mitochondrial DNA sequences retrieved from the nucleotide database in GeneBank (accession number of the sequences can be found in the supplementary file (Supplementary Table S7)). A complete sequenced genome of *Heterodontus portusjacksoni* was not available in the database, therefore, two other species of *Heterodontus* (*H*. *francisci* and *H*. *zebra*) were used as an outgroup of the clade consisting of [Orectolobiformes + [Carcharhiniformes + Lamniformes]. The sequences were aligned in MAFFT^69^ and a matrix of approximately 16.5kbp resulted after trimming the edges. To construct the phylogeny the GTR + G + I substitution model was employed. The alignment was used in BEAST 2 software^70^, two parallel MCMC runs were performed over 10,000,000 generations sampling every 1000 generations.10% of the generations were set as a burn-in on TreeAnnotator^70^ to obtain a maximum credibility tree on which the topology was used for stochastic character mapping with the make.simmap function in phytools^71^ to perform an ancestral state reconstruction on the tooth histotype. Data for the tooth histology of the 26 species was retrieved from this study and the literature^22,26,31,34,43,44,49^. The final tree was edited in FigTree (v. 1.4.4).

## Supplementary information

Supplementary Material

## Data Availability

All specimens are deposited in either of the following collections and are publicly accessible: 1. collection of the Department of Palaeontology, University of Vienna, Vienna, Austria; 2. Haimuseum und Sammlung R. Kindlimann, Aathal-Seegräben, Switzerland; 3. Jura Museum Eichstätt, Germany. Micro-CT scans are stored at the Department of Palaeontology, University of Vienna, Vienna, Austria. All data generated and analyzed during this study are included in this published article (and its Supplementary Information files). Detailed deposition information can be found in Supplementary Table S5.
