# Supplementary material for: Micro-computed tomography imaging reveals the development of a unique tooth mineralization pattern in mackerel sharks (Chondrichthyes; Lamniformes) in deep time
Source: Sci Rep. 2019 Jul 4;9:9652. doi: 10.1038/s41598-019-46081-3 (PMC6609643; doi:10.1038/s41598-019-46081-3)
Supplement: Supplementary file 1 — Supplementary Material [file 41598_2019_46081_MOESM1_ESM.pdf]

Micro-computed tomography imaging reveals the development of a unique tooth mineralization pattern in mackerel sharks (Chondrichthyes; Lamniformes) in deep time.

Patrick L. Jambura[1*], René Kindlimann[2], Faviel López-Romero[1], Giuseppe Marramà[1], Cathrin Pfaff[1], Sebastian Stumpf[1], Julia Türtscher[1], Charlie J. Underwood[3], David J. Ward[4], Jürgen Kriwet[1]

Affiliations

[1]Department of Palaeontology, University of Vienna, Vienna, Austria

[2]Haimuseum und Sammlung R. Kindlimann, Aathal-Seegräben, Switzerland

[3]Department of Earth and Planetary Sciences, Birkbeck, University of London, London, UK

[4]Department of Earth Sciences, Natural History Museum, London, UK

Corresponding Author

*patrick.jambura@gmail.com

Short Title

Evolution of the tooth mineralization in lamniform sharks

Supplementary Information

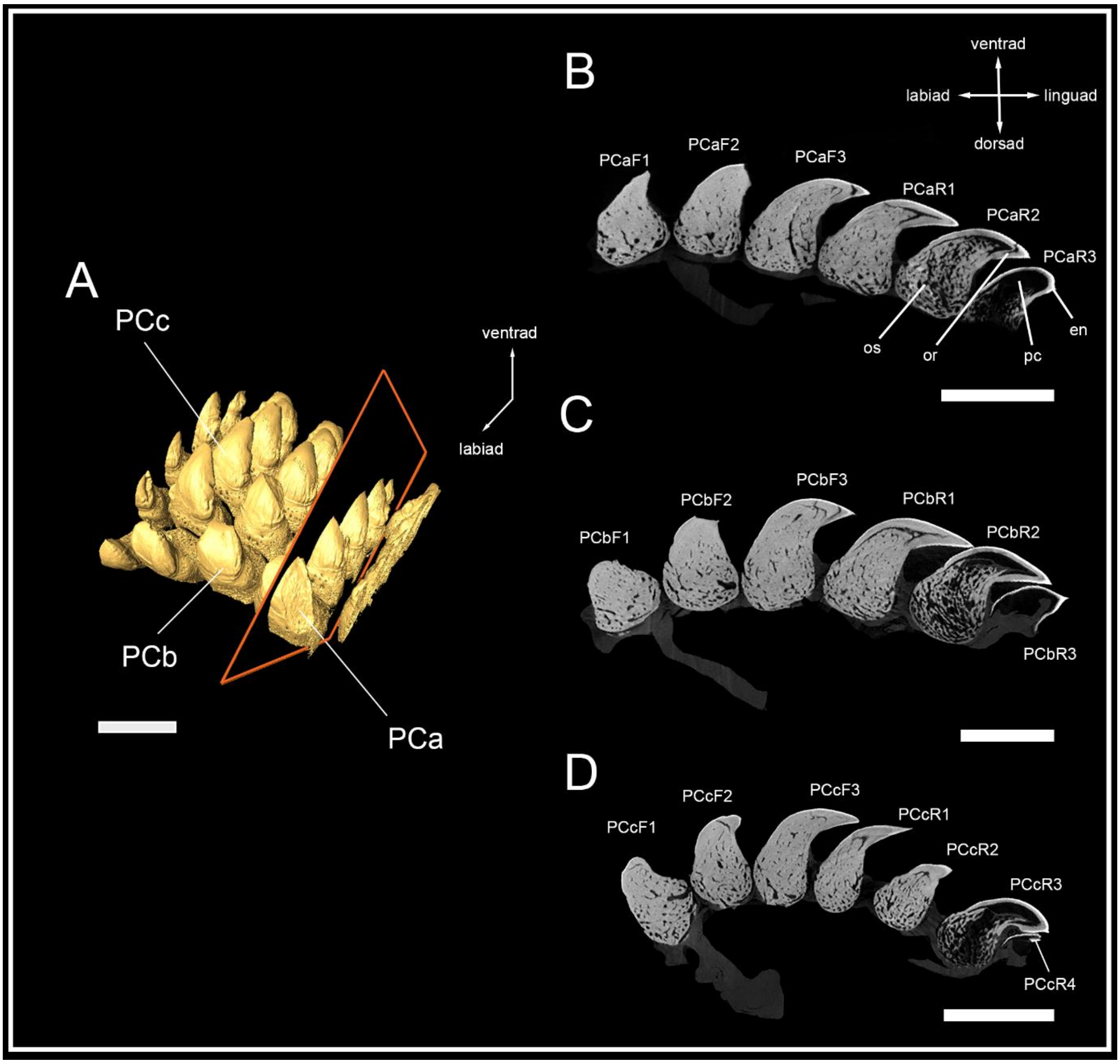

Figure S1. Virtual tooth file sections of *Cetorhinus maximus*. (A) isosurface; (B) first tooth file (Pca); (C) second tooth file (PCb); (D) third tooth file (PCc). en, enameloid; F1, first functional tooth; F2, second functional tooth; F3, third functional tooth; R1, first replacement tooth; R2, second replacement tooth; R3, third replacement tooth; R4, fourth replacement tooth; or, orthodentine; os, osteodentine; pc, pulp cavity; PC, palatoquadrate cartilage; scalebar = 0.5 cm.

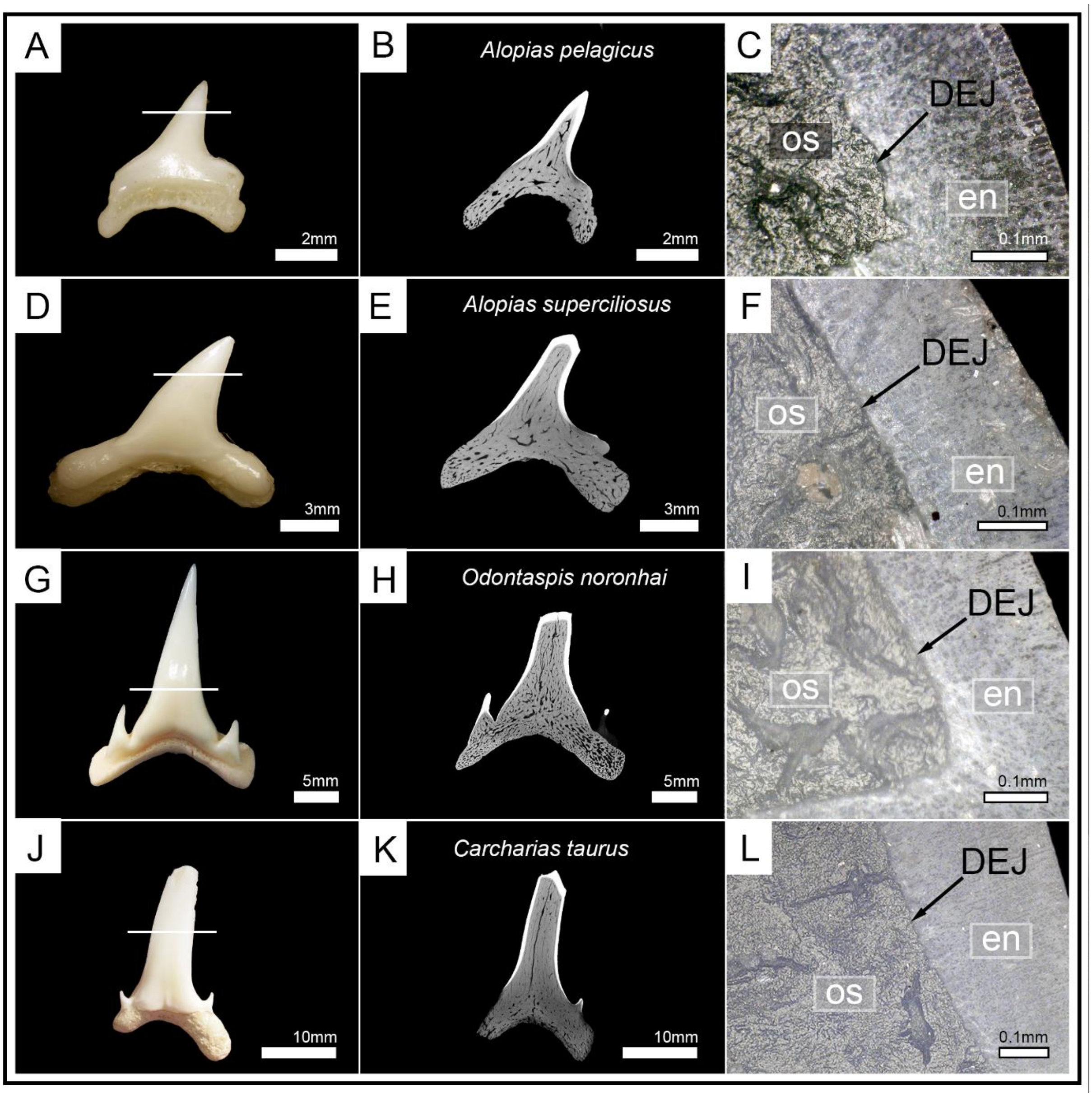

Figure S2. Micro-CT images and tooth sections of extant lamniform sharks. (A-C) *Alopias pelagicus* (EMRG-Chond-T-25); (D-F) *Alopias superciliosus* (EMRG-Chond-T-26); (G-I) *Odontaspis noronhai* (EMRG-Chond-T-3); (J-L) *Carcharias taurus* (EMRG-Chond-T-5); white lines through the tooth indicate the plane of the tooth section; en, enameloid; os, osteodentine.

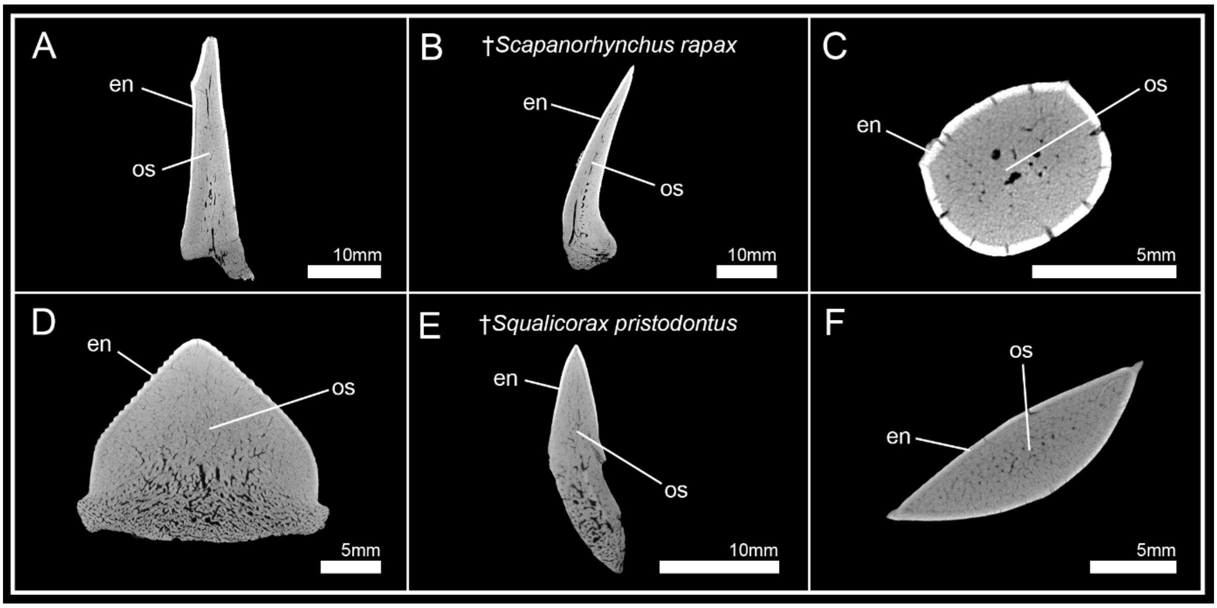

Figure S3. Micro-CT images of extinct lamniform sharks in frontal, sagittal, and axial view: (A-C) †*Scapanorhynchus rapax* (EMRG-Chond-T-49); (D-F) †*Squalicorax pristodontus* (EMRG-Chond-T-46); en, enameloid; os, osteodentine.

Table S1. Synopsis of the tooth histology of fossil and extant lamniform sharks. Only publications providing photographs or CT-images with proper quality to unambiguously show the presence or absence of orthodentine were considered here.

Species	Family	Time	Histology	Source
Alopias pelagicus	Alopiidae	recent	osteodont	Fig. S1 online
Alopias superciliosus	Alopiidae	recent	osteodont	Fig. S1 online; Schnetz et al. 2016
Alopias vulpinus	Alopiidae	recent	osteodont	Fig. 3
Carcharias taurus	Odontaspididae	recent	osteodont	Fig. S1 online
Carcharodon carcharias	Lamnidae	recent	osteodont	Goto 1999; Moyer et al 2015a; Moyer et al. 2015b; Schnetz et al. 2016
Cetorhinus maximus	Cetorhinidae	recent	pseudoosteodont	Figs 1 & 2
Isurus oxyrinchus	Lamnidae	recent	osteodont	Schnetz et al. 2016; Jambura et al. 2018
Lamna ditropis	Lamnidae	recent	osteodont	Kazikawa 1984
Lamna nasus	Lamnidae	recent	osteodont	Fig; 3; Moyer et al. 2015b; Schnetz et al. 2016
Megachasma pelagios	Megachasmidae	recent	osteodont	Yabumoto et al. 1997; Goto 1999
Megachasma pelagios	Megachasmidae	Pliocene, Neogene	osteodont	Fig 4
Mitsukurina owstoni	Mitsukurinidae	recent	osteodont	Fig 3
Odontaspis ferox	Odontaspididae	recent	osteodont	Fig. 3
Odontaspis noronhai	Odontaspididae	recent	osteodont	Fig. S1 online
Pseudocarcharias kamoharai	Pseudocarchariidae	recent	osteodont	Figs. 1 & 2
†*Otodus megalodon*	Otodontidae	Miocene, Neogene	osteodont	Fig 5
†*Cretalamna appendiculata*	Cretoxyrhinidae?	Cretaceous	osteodont	Schnetz et al. 2016
†*Dwardius woodwardi*	inc. sed.	Albian, Cretaceous	osteodont	Fig 5
†*Leptostyrax sp.*	Eoptolamnidae	Albian/Cenomanian, Cretaceous	osteodont	Fig 4
†*Palaeocarcharias stromeri*	inc. sed.	Tithonian, Jurassic	osteodont	Fig 6; de Beaumont 1960, Landemaine et al. 2018
†*Palaeocarcharodon orientalis*	Lamniformes incertae fam.	Eocene, Paleogene	osteodont	Fig 4
†*Protolamna sokolovi*	Eoptolamnidae	Albian, Cretaceous	osteodont	Schnetz et al. 2016
†*Scapanorhynchus rapax*	Mitsukurinidae	Maastrichtian, Cretaceous	osteodont	Fig. S2 online
†*Squalicorax curvatus*	Anacoracidae	Cretaceous	osteodont	Hoffman et al. 2016
†*Squalicorax primaevus*	Anacoracidae	Albian, Cretaceous	osteodont	Fig 5
†*Squalicorax pristodontus*	Anacoracidae	Maastrichtian, Cretaceous	osteodont	Fig. S2 online;
†*Striatolamia macrota*	Odontaspididae	Paleocene-Eocene	osteodont	Møller et al. 1975
†*Truyolsodontos estauni*	Truyolsodontidae	Cenomanian, Cretaceous	osteodont	Bernardez 2018

Table S2. Tooth mineralization sequence of the upper jaws of the basking shark (*Cetorhinus maximus* (7-692/RZ)) and the crocodile shark (*Pseudocarcharias kamoharai* (7-693/RZ)). Colours indicate similar mineralization stages.

	Tooth	enameloid	orthodentine	pulp cavity	root
Cetorhinus maximus	PCaF1	complete	fully mineralized	cavity fully filled	fully mineralized
	PCaF2	complete	fully mineralized	cavity fully filled	fully mineralized
	PCaF3	complete	fully mineralized	cavity fully filled	fully mineralized
	PCaR1	complete	fully mineralized	cavity partly filled	fully mineralized
	PCaR2	complete	partly mineralized	cavity partly filled	partly mineralized
	PCaR3	complete	partly mineralized	cavity partly filled	partly mineralized
Pseudocarcharias kamoharai	LPC1F1	complete	Absent	cavity fully filled	fully mineralized
	LPC1F2	complete	Absent	cavity fully filled	fully mineralized
	LPC1R1	complete	Absent	cavity fully filled	fully mineralized
	LPC1R2	complete	Absent	cavity partly filled	partly mineralized
	LPC1R3	complete	Absent	cavity partly filled	partly mineralized
	LPC1R4	complete	Absent	Hollow	absent
	LPC1R5	incomplete	Absent	Hollow	absent
	LPC1R6	incomplete	Absent	Hollow	absent

Table S3. Tooth mineralization sequence in the crocodile shark (*Pseudocarcharias kamoharai*). L, left; PC, palatoquadrate cartilage; F, functional tooth; R, replacement tooth

Pseudocarcharias kamoharai

	tooth	enameloid	orthodentine	pulp cavity	root
	LPC1F1	complete	absent	cavity fully filled	fully mineralized
	LPC1F2	complete	absent	cavity fully filled	fully mineralized
	LPC1R1	complete	absent	cavity fully filled	fully mineralized
	LPC1R2	complete	absent	cavity partly filled	partly mineralized
	LPC1R3	complete	absent	cavity partly filled	partly mineralized
	LPC1R4	complete	absent	hollow	absent
	LPC1R5	incomplete	absent	hollow	absent
	LPC1R6	incomplete	absent	hollow	absent
	LPC2F1	complete	absent	cavity fully filled	fully mineralized
	LPC2F2	complete	absent	cavity fully filled	fully mineralized
	LPC2R1	complete	absent	cavity fully filled	fully mineralized
	LPC2R2	complete	absent	cavity partly filled	partly mineralized
	LPC2R3	complete	absent	cavity partly filled	partly mineralized
	LPC2R4	complete	absent	hollow	absent
	LPC2R5	incomplete	absent	hollow	absent
	LPC2R6	incomplete	absent	hollow	absent
	LPC3R1	complete	absent	cavity fully filled	fully mineralized
	LPC3R2	complete	absent	cavity partly filled	partly mineralized
	LPC3R3	complete	absent	cavity partly filled	partly mineralized
	LPC3R4	complete	absent	hollow	absent
	LPC3R5	incomplete	absent	hollow	absent
	LPC4F1	complete	absent	cavity fully filled	fully mineralized
	LPC4R1	complete	absent	cavity fully filled	fully mineralized
	LPC4R2	complete	absent	cavity partly filled	partly mineralized
	LPC4R3	complete	absent	hollow	absent
	LPC4R4	incomplete	absent	hollow	absent
	LPC4R5	incomplete	absent	hollow	absent

Table S4. Tooth mineralization sequence in the basking shark (*Cetorhinus maximus*).
L, left; PC, palatoquadrate cartilage; F, functional tooth; R, replacement tooth

Cetorhinus maximus

tooth	enameloid	orthodentine	pulp cavity	root
PCaF1	complete	fully mineralized	cavity fully filled	fully mineralized
PCaF2	complete	fully mineralized	cavity fully filled	fully mineralized
PCaF3	complete	fully mineralized	cavity fully filled	fully mineralized
PCaR1	complete	fully mineralized	cavity partly filled	fully mineralized
PCaR2	complete	partly mineralized	cavity partly filled	partly mineralized
PCaR3	complete	partly mineralized	cavity partly filled	partly mineralized
PCbF1	complete	fully mineralized	cavity fully filled	fully mineralized
PCbF2	complete	fully mineralized	cavity fully filled	fully mineralized
PCbF3	complete	fully mineralized	cavity fully filled	fully mineralized
PCbR1	complete	fully mineralized	cavity partly filled	fully mineralized
PCbR2	complete	partly mineralized	cavity partly filled	partly mineralized
PCbR3	complete	absent	hollow	absent
PCcF1	complete	fully mineralized	cavity fully filled	fully mineralized
PCcF2	complete	fully mineralized	cavity fully filled	fully mineralized
PCcF3	complete	fully mineralized	cavity fully filled	fully mineralized
PCcF4	complete	fully mineralized	cavity fully filled	fully mineralized
PCcR1	complete	fully mineralized	cavity partly filled	partly mineralized
PCcR2	complete	partly mineralized	cavity partly filled	partly mineralized
PCcR3	incomplete	absent	hollow	absent

Table S5. Examined jaws and teeth of extinct and extant lamniform sharks. JME-SOS-2294 is the holotypic specimen of †*Palaeocarcharias stromeri* and is deposited in the collection of the Jura-Museum Eichstätt. Specimens with accession numbers starting with Inv.nr are deposited in the collection of the Haimuseum und Sammlung R. Kindlimann. The remaining specimens are deposited in the collection of the Department of Palaeontology at the University of Vienna. All specimens are publicly accessible.

Species	Family	Material	Accession number	Age	Locality
†*Otodus megalodon*	Otodontidae	Tooth	Emrg-chond-t-57	Miocene, neogene (5-23 mya)	No detailed locality information, usa
†*Dwardius woodwardi*	Inc. Sed.	Tooth	Emrg-chond-t-53	Albian, cretaceous (101-113 mya)	Gault clay formation, uk
†*Leptostyrax* sp.	Eoptolamnidae	Tooth	Inv.nr. 7-690	Albian/cenomanian, cretaceous (94-113 mya)	Kem kem beds, morocco
†*Palaeocarcharias stromeri*	Inc. Sed.	Tooth	Sos_2294_22_2	Tithonian, jurassic (145-152 mya)	Eichstätt, germany
†*Palaeocarcharodon orientalis*	Lamniformes incertae fam.	Tooth	Emrg-chond-t-50	Eocene, paleogene (34-56)	Khouribga, morocco
†*Scapanorhynchus rapax*	Mitsukurinidae	Tooth	Emrg-chond-t-49	Maastrichtian, cretaceous (66-72 mya)	Khouribga, morocco
†*Squalicorax primaevus*	Anacoracidae	Tooth	Emrg-chond-t-54	Albian, cretaceous (101-113 mya)	Naccolt, uk
†*Squalicorax pristodontus*	Anacoracidae	Tooth	Emrg-chond-t-46	Maastrichtian, cretaceous (66-72 mya)	No detailed locality information, morocco
Alopias pelagicus	Alopiidae	Tooth	Emrg-chond-t-25	Recent	Philippines
Alopias superciliosus	Alopiidae	Tooth	Emrg-chond-t-26	Recent	Indian ocean
Alopias vulpinus	Alopiidae	Tooth	Emrg-chond-t-27	Recent	North sea
Carcharias taurus	Odontaspididae	Tooth	Emrg-chond-t-5	Recent	Aquarium la rochelle, france
Cetorhinus maximus	Cetorhinidae	Jaw	Inv.nr. 7-692/rz	Recent	China
Cetorhinus maximus	Cetorhinidae	Tooth	Emrg-chond-t-24	Recent	No locality information
Lamna nasus	Lamnidae	Tooth	Emrg-chond-t-4	Recent	Scottish north sea
Megachasma pelagios	Megachasmidae	Tooth	Emrg-chond-t-44	Pliocene, neogene (2-5 mya)	Bahia ingles formaton, chile
Mitsukurina	Owstoni	Tooth	Emrg-chond-t-1	Recent	Philippines
Odontaspis ferox	Odontaspididae	Tooth	Emrg-chond-t-2	Recent	Taiwan
Odontaspis noronhai	Odontaspididae	Tooth	Emrg-chond-t-3	Recent	Indian ocean
Pseudocarcharias kamoharai	Pseudocarchariidae	Jaw	Inv.nr. 7-693/rz	Recent	Pacific
Pseudocarcharias kamoharai	Pseudocarchariidae	Tooth	Emrg-chond-t-28	Recent	Philippines

Table S6. Applied settings for the micro-CT scanner Bruker SkyScan1173 (Kontich, Belgium)

Species	Material	Age	Inventory-no	Voxel size [µm]	Source-voltage [kv]	Source current [µa]	Filter	Exposure time [ms]	Rotation step [°]	Frame averaging
†*Otodus megalodon*	tooth	Neogene	EMRG-Chond-T-57	30,042382	130	61	brass 0.25mm	1249	0,2	3
†*Dwardius woodwardi*	tooth	Cretaceous	EMRG-Chond-T-53	17,882692	100	80	Al 1.0mm	750	0,2	3
†*Leptostyrax* sp.	tooth	Cretaceous	Inv.nr. 7-690	17,168527	100	80	Al 1.0mm	750	0,2	3
†*Palaeocarcharias stromeri*	tooth	Jurassic	SOS_2294_22_2	6,438884	80	100	no Filter	650	0,2	3
†*Palaeocarcharodon orientalis*	tooth	Paleogene	EMRG-Chond-T-50	23,247514	100	80	Al 1.0mm	750	0,2	3
†*Scapanorhynchus rapax*	tooth	Cretaceous	EMRG-Chond-T-49	19,695573	100	80	Al 1.0mm	750	0,2	3
†*Squalicorax primaevus*	tooth	Cretaceous	EMRG-Chond-T-54	7,868931	50	100	User Filter	1100	0,2	3
†*Squalicorax pristodontus*	tooth	Cretaceous	EMRG-Chond-T-46	17,882692	100	80	Al 1.0mm	750	0,2	3
Alopias pelagicus	tooth	recent	EMRG-Chond-T-25	6,438884	80	100	no Filter	650	0,2	3
Alopias superciliosus	tooth	recent	EMRG-Chond-T-26	7,511849	80	100	no Filter	650	0,2	3
Alopias vulpinus	tooth	recent	EMRG-Chond-T-27	7,511849	80	100	no Filter	650	0,2	3
Carcharias taurus	tooth	recent	EMRG-Chond-T-5	12,87667	80	100	no Filter	650	0,2	3
Cetorhinus maximus	jaw	recent	Inv.nr. 7-692/RZ	15,022599	100	80	Al 1.0mm	750	0,2	5
Cetorhinus maximus	tooth	recent	EMRG-Chond-T-24	6,438884	80	100	no Filter	650	0,2	3
Lamna nasus	tooth	recent	EMRG-Chond-T-4	12,160788	80	100	no Filter	650	0,2	3
Megachasma pelagios	tooth	Neogene	EMRG-Chond-T-44	11,087824	100	80	Al 1.0mm	750	0,2	3
Mitsukurina	tooth	recent	EMRG-Chond-T-1	13,894228	80	100	no Filter	650	0,2	3
Odontaspis ferox	tooth	recent	EMRG-Chond-T-2	13,894228	80	100	no Filter	650	0,2	3
Odontaspis noronhai	tooth	recent	EMRG-Chond-T-3	13,894228	80	100	no Filter	650	0,2	3
Pseudocarcharias kamoharai	jaw	recent	Inv.nr. 7-693/RZ	23,963395	100	80	Al 1.0mm	750	0,2	3
Pseudocarcharias kamoharai	tooth	recent	EMRG-Chond-T-28	7,868931	80	100	no Filter	650	0,2	3

Table S7. Accession numbers of the mitochondrial DNA sequences used to build the tree.

Species	GenBank ID
Alopias pelagicus	KF020876.1
Alopias superciliosus	KC757415.1
Alopias vulpinus	MF374733.1
Carcharhinus leucas	KF646785.1
Carcharhinus melanopterus	KJ720818.1
Carcharhinus obscurus	KC470543.1
Carcharhinus plumbeus	KJ740750.1
Carcharias taurus	MH823675.1
Carcharodon carcharias	KY067590.1
Cetorhinus maximus	KM096988.1
Galeocerdo cuvier	KF111728.1
Ginglymostoma cirratum	KU904394.1
Hemigaleus microstoma	KT003687.1
Hemipristis elongata	KU508621.1
Heterodontus francisci	AJ310141.1
Heterodontus zebra	KC845548.1
Isurus oxyrinchus	KF361861.1
Lamna ditropis	KF962053.1
Lamna nasus	KX610464.1
Megachasma pelagios	KC702506.1
Mitsukurina owstoni	EU528659.1
Nebrius ferrugineus	KT852575.1
Prionace glauca	KF356249.1
Pseudocarcharias kamoharai	KM575726.1
Rhincodon typus	KC633221.1
Sphyrna zygaena	KM489157.1
Stegostoma fasciatum	KU057952.1

Supplementary Discussion S1: The phylogenetic relationships of †*Palaeocarcharias stromeri*

Despite the complete nature of the fossil specimens, only a few studies attempted to clarify the relationships of †*Palaeocarcharias*. In the original description (De Beaumont 1960) and subsequent literature (Cappetta, 1987, 2012; Duffin 1988) †*P. stromeri* was considered as basal lamniform or in any case related to lamniform sharks, or alternatively as member of orectolobiforms (Duffin 1988), or as a transitional form between orectolobiforms and lamniforms (Applegate 2001). However, the phylogenetic position of †*Palaeocarcharias* has never been tested using cladistics method until recently, when Landemaine et al. (2018) included this taxon in a phylogenetic framework employing Bayesian methodologies. The phylogenetic analysis of Landemaine et al. (2018) was based on a dataset of 198 morphological characters, 169 of which were taken directly from the dataset of Klug (2010), 21 from Shirai (1996) and eight newly introduced. The ingroup taxa of Landemaine et al. (2018) included almost all the ingroup taxa used by Klug (2010) representing all major elasmobranch orders with the inclusion of the †Synechodontiformes and the †Hybodontiformes, the latter used as outgroup by Klug (2010) and replaced with Rajiformes by Landemaine et al. (2018). The analysis of Landemaine et al. (2018) detected †*Palaeocarcharias* nested within the galeomorph sharks and as sister to the pair formed by Lamniformes plus Carcharhiniformes and sharing with them two tooth characters: holaulacorhizous vascularization pattern and the absence of a lingual tooth uvula.

However, our crosschecking of the data matrix of Landemaine et al. (2018) executed with WinClada 1.00.08 (Nixon 2002) detected a significant amount of uninformative characters (62, corresponding to 31.3% of the total). We then rerun the analysis using the remaining 136 informative characters (see the new character list below). The new matrix was compiled in MESQUITE v.3.03 (Maddison & Maddison, 2008) and the phylogenetic analysis was performed with WinClada and TNT 1.5 using several methods (branch-and-bound, heuristic search, etc) (Nixon 2002; Goloboff et al., 2008). All characters are here considered unordered and given equal weight.

The analysis of 136 morphological characters coded for 20 taxa produced always, even with different methods, a single most parsimonious tree (MPT) whose length is shorter (TL=278) than using the complete character list (TL=330) (Supplementary Fig. S4 online). The phylogenetic hypothesis is supported by the following indices: consistency index of 0.61, and retention index of 0.74. The retrieved relationships positions of †*Palaeocarcharias* within the phylogenetic tree of all our analyses are not consistent with those of Landemaine et al. (2018). In fact, our analysis detected †*Palaeocarcharias* as basal to all living galeomorph sharks, sister to (Carcharhiniformes+Lamniformes) + (Orectolobiformes+*Heterodontus*). Detection of

†*Palaeocarcharias* as basal to all galeomorphs is probably related to the fact the analysis failed to detect unambiguous synapomorphies uniting †*Palaeocarcharias* to any of the living galeomorph sharks. In fact, Landemaine et al. (2018) did not include most of the cranial or postcranial skeletal characters for †*Palaeocarcharias*, resulting in 65% of missing characters for this taxon in their matrix. Excluding phylogenetic uninformative characters, it resulted even in 68% of missing characters in our matrix. Currently, this analysis therefore cannot resolve the phylogenetic relationships of †*Palaeocarcharias* beyond doubt until a robust comprehensive morphological analysis including tooth, cranial and postcranial skeletal characters will be provided.

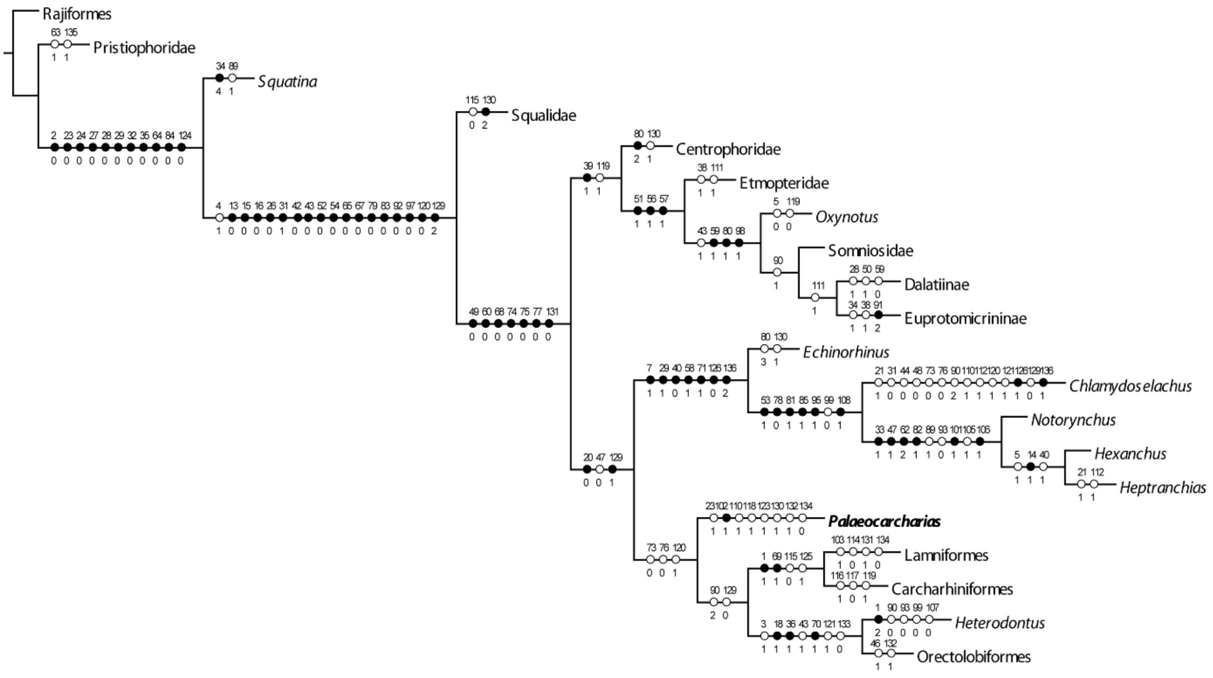

Figure S4. The single tree retrieved using both TNT and WinClada softwares and based on 136 morphological characters and 20 taxa, showing the hypothetic relationships of †*Palaeocarcharias*. The white circles are the homoplastic characters. The numbers above the circles are the characters of the list in Supplementary Note S1, the numbers below are character states.

Supplementary Note S1: Modified character list

Here below is the modified character list after the exclusion of the phylogenetic uninformative characters of Landmaine et al. (2018) (i.e., their chs. 20, 23, 31, 33, 36-39, 44, 46, 49-51, 68, 71, 76, 77, 79, 80, 87-89, 91, 98, 102-105, 110, 116, 117, 119, 122, 126, 137, 138, 141, 147, 157, 159, 160, 162, 165-167, 169-177, 179, 181, 182, 186, 187, 194, 197, 198).

1. rostral process
2. precerebral fossa
3. ethmoidal region of neurocranium
4. ethmoidal canal
5. subnasal fenestra
6. superficial ophthalmic nerve
7. ectethmoid process
8. orbital articulation
9. suborbital shelf
10. basitrabecular process
11. postorbital articulation
12. hyomandibular fossa in the posterior part
13. hyomandibular fossa not composed
14. concavity ventral to hyomandibular fossa
15. basioccipital fovea
16. occipital hemicentrum
17. beta-slip of obliquus
18. nasoral groove
19. mesonarial flap
20. spiracle valve
21. basihyal elongated
22. internal lobe of anterior
23. basihyal about
24. preorbital canal
25. ethmoidal nerve
26. exit of ethmoidal canal
27. entrance of ethmoidal canal
28. profundus canal
29. antorbital (ectethmoid) process
30. eye-stalk

31. basal angle
32. rostral process
33. hyoid arch
34. genio-coracoideus
35. uncovered precerebral fossa
36. nasal capsule almost attached to orbit
37. craniopalatine articulation
38. rectus externus
39. suborbitalis muscle
40. lateral line sensory canal
41. absence of extrabranchial cartilages
42. adductor mandibulae not divided
43. suborbitalis inserting on adductor mandibulae
44. adductor mandibulae superficialis absent
45. adductor mandibulae superficialis inserting on subcutaneous tissue
46. levator palatoquadrati muscle not separated from spiracularis
47. constrictor hyoideus dorsalis inserting on hyomandibula
48. arcualis dorsalis composed of single slip
49. supraneurals rudimentary
50. interpharyngobranchialis present
51. genio-coracoideus originating on fascia-covering coraco-arcualis
52. coraco-hyomandibularis muscle absent
53. three columns of heart valves
54. inclinator dorsalis muscle
55. flexor caudalis muscle restricted
56. luminous organs absent
57. parietalis muscle present
58. suborbitalis not arising from antorbital cartilage
59. suborbitalis inserting on lower jaw through
60. suborbitalis not originating from suborbital surface
61. adductor mandibulae superficialis with one insertion dorsally
62. constrictor hyoideus
63. subspinalis externus absent
64. coraco-hyomandibularis absent
65. ventral bundle musculature of caudal region
66. flexor caudalis muscles not expanding anteriorly
67. inclinator dorsalis muscle with origins

68. plate-like supraneurals
69. suborbitalis muscle not originating posterior aspect of nasal capsule
70. suborbitalis muscle not originating on upper preorbital wall
71. notochord constricted along entire vertebral column
72. suborbitalis muscle originating on suborbital shelf
73. hemal spines not forming complete arches
74. precaudal hemal process not elongated
75. precaudal longitudinal ridges
76. hemal arches poorly developed
77. basiventral process of precaudal region
78. coracoids fused
79. single facet for the articulation
80. pectoral basals separated into three distinct cartilages
81. perctoral propterygium articulating with radials
82. short proximal segment of metapterygium
83. single articular condyle on pectoral girdle
84. separate condyle for propterygium
85. pectoral propterygium with radials
86. propterygium not directed anteriorly
87. ventral marginal cartilage of clasper
88. pelvic girdle (puboischiadic bar) not very elongated
89. anterior pelvic basal
90. dorsal fin endoskeleton
91. both dorsal fins with triangular
92. basal plates not anchored
93. anal fin skeleton composed of basal cartilage and radials
94. anal fin present
95. sixth branchial unit
96. seventh branchial unit
97. anterior basibranchial
98. hypobranchial bar
99. posteriormost elements of dorsal gill arches
100. pharyngobranchial blade
101. paired basibranchials
102. tooth crown devoid of an apron
103. less than two dorsal labial cartilages
104. ventral labial cartilage

105. less than one ventral labial cartilage
106. dorsal and ventral labial cartilages not fused
107. dorsal fin spines present
108. number of dorsal fins
109. large paired fins
110. small and round tips of paired fins
111. rounded and small dorsal fins
112. longitudinal extension of base
113. clutching-type dentition
114. tearing-type dentition
115. cutting-type dentition
116. cutting-clutching-type dentition
117. crushing-type dentition
118. grinding-type dentition
119. dignathic heterodonty
120. anaulacorhizouse type
121. hemiaulacorizous
122. holaulacorhizous
123. lateral cusplets
124. coracobranchialis 1 inserting on the basihyal and/or hyoid cartilage
125. pectoral fin radials aplesodic
126. primary calcification of vertebrae
127. secondary calcification of vertebrae
128. dorsal fin dual
129. lower jaw teeth arranged diagonally
130. upper jaw teeth arranged diagonally
131. precaudal keel absent
132. dermal cephalic lobe
133. tooth crown with uvula
134. crown with labio-basal coronal extension
135. tooth roots with only one median labio-lingo canal
136. outline of the basal root face

Table S8. Modified character matrix

	1	2	3	4	5	6	7	8	9	10	11	12	13	14	15	16	17	18	19	20	21	22	23	24	25	26	27	28	29	30
Rajiformes	0	1	1	2	0	0	3	0	1	0	0	0	1	0	1	1	0	0	1	1	0	1	1	1	1	1	1	1	2	0
Heterodontus	2	0	1	0	0	0	0	0	0	0	0	1	0	0	0	0	0	1	0	0	0	0	0	0	0	?	?	0	0	0
Orectolobiformes	0	0	1	0	0	1	0	0	0	0	0	1	0	0	0	0	0	1	0	0	0	0	0	0	0	?	?	0	0	0
Lamniformes	1	0	0	0	0	1	0	0	0	0	0	1	0	0	0	0	0	0	0	0	0	0	0	0	0	?	?	0	0	0
Carcharhiniformes	1	0	0	0	0	1	0	0	0	0	0	1	0	0	0	0	0	0	0	0	0	0	0	0	0	?	?	0	0	0
Chlamydoselachus	0	0	0	0	0	0	1	1	1	1	1	0	0	0	0	0	0	0	0	0	1	0	0	0	0	?	?	0	1	0
Hexanchus	0	0	0	1	1	0	1	1	1	1	2	0	0	1	0	1	0	0	0	0	0	0	0	0	1	0	0	0	1	0
Notorynchus	0	0	0	0	0	0	1	1	1	1	2	0	0	0	0	1	0	0	0	0	0	0	0	0	0	?	?	0	1	0
Heptranchias	0	0	0	1	1	0	1	1	1	1	2	0	0	1	0	1	0	0	0	0	1	0	0	0	1	0	0	0	1	0
Echinorhinus	0	0	0	1	0	0	1	1	1	1	0	0	0	0	0	1	0	0	1	0	0	1	0	0	1	0	0	0	1	0
Etmopteridae	0	0	0	1	1	0	0	1	1	1	0	0	0	0	0	0	0	0	1	1	0	1	0	0	1	0	0	0	0	1
Somniosidae	0	0	0	1	1	0	0	1	1	1	0	0	0	0	0	0	0	0	1	1	0	1	0	0	1	0	0	0	0	0
Oxynotus	0	0	0	1	0	0	0	1	1	1	0	0	0	0	0	0	0	0	1	1	0	1	0	0	1	0	0	0	0	1
Dalatiinae	0	0	0	1	1	0	0	1	1	1	0	0	0	0	0	0	0	0	1	1	0	1	0	0	1	0	0	1	0	0
Euprotomicrininae	0	0	0	1	1	0	0	1	1	1	0	0	0	0	0	0	0	0	1	1	0	1	0	0	1	0	0	0	0	0
Centrophoridae	0	0	0	1	1	0	0	1	1	1	0	0	0	0	0	0	0	0	1	1	0	1	0	0	1	0	0	0	0	0
Squalidae	0	0	0	1	1	0	0	1	1	1	0	0	0	0	0	0	0	0	1	1	0	1	0	0	1	0	0	0	0	0
Squatina	0	0	0	2	0	0	0	1	1	1	0	0	1	0	1	1	1	0	1	1	0	1	0	0	1	1	0	0	0	0
Pristiophoridae	0	1	0	2	0	0	2	1	1	1	0	0	1	0	1	1	1	0	1	1	0	1	1	1	1	1	1	1	2	0
†*Palaeocarcharias*	?	?	?	?	?	?	?	?	?	?	?	?	?	?	?	?	?	?	?	?	?	?	1	?	?	?	?	?	?	?

	31	32	33	34	35	36	37	38	39	40	41	42	43	44	45	46	47	48	49	50	51	52	53	54	55	56	57	58	59	60
Rajiformes	0	1	0	0	1	0	2	0	0	1	2	1	2	1	2	1	0	0	1	1	0	1	0	1	2	0	0	0	0	1
Heterodontus	0	0	0	0	0	1	0	0	0	1	1	0	1	1	0	0	0	0	0	0	0	0	0	0	0	0	0	0	0	0
Orectolobiformes	0	0	0	0	0	1	0	0	0	1	1	?	1	0	?	1	0	0	0	0	0	0	0	0	0	0	0	0	0	0
Lamniformes	0	0	0	0	0	0	0	0	0	1	1	0	0	0	?	0	0	0	0	0	0	0	0	0	0	0	0	0	0	0
Carcharhiniformes	0	0	0	0	0	0	0	0	0	1	1	0	0	0	?	0	0	0	0	0	0	0	0	0	0	0	0	0	0	0
Chlamydoselachus	0	0	0	0	0	0	1	0	0	0	0	0	0	0	?	0	0	0	0	0	0	0	1	0	0	0	0	1	0	0
Hexanchus	1	0	1	0	0	0	1	0	0	1	1	0	0	1	1	0	1	1	0	0	0	0	1	0	0	0	0	1	0	0
Notorynchus	1	0	1	0	0	0	1	0	0	0	1	0	0	1	1	0	1	1	0	0	0	0	1	0	0	0	0	1	0	0
Heptranchias	1	0	1	0	0	0	1	0	0	1	1	0	0	1	1	0	1	1	0	0	0	0	1	0	0	0	0	1	0	0
Echinorhinus	1	0	0	0	0	0	1	0	0	0	2	0	0	1	3	0	0	1	0	0	0	0	0	0	0	0	0	1	0	0
Etmopteridae	1	0	0	1	0	0	1	1	1	1	2	0	0	1	0	0	2	1	0	0	1	0	0	0	0	1	1	0	0	0
Somniosidae	1	0	0	2	0	0	1	0	1	1	2	0	1	1	0	0	2	1	0	0	1	0	0	0	0	1	1	0	1	0
Oxynotus	1	0	0	2	0	0	1	0	1	1	2	0	1	1	0	0	2	1	0	0	1	0	0	0	0	1	1	0	1	0
Dalatiinae	1	0	0	2	0	0	1	0	1	1	2	0	1	1	0	0	2	1	0	1	1	0	0	0	0	1	1	0	0	0

Euprotomicrininae	1	0	0	1	0	0	1	1	1	1	2	0	1	1	0	0	2	1	0	0	1	0	0	0	0	1	1	0	1	0
Centrophoridae	1	0	0	0	0	0	1	0	1	1	2	0	0	1	0	0	2	1	0	0	0	0	0	0	0	0	0	0	0	0
Squalidae	1	0	0	0	0	0	1	0	0	1	2	0	0	1	0	0	2	1	1	0	0	0	0	0	1	0	0	0	0	1
Squatina	0	0	0	4	0	0	1	0	?	1	2	1	2	1	0	0	2	1	1	0	0	1	0	1	2	0	0	?	?	?
Pristiophoridae	0	1	0	0	1	0	1	0	0	1	2	1	2	1	0	0	2	1	1	0	0	1	0	1	2	0	0	0	0	1
†*Palaeocarcharias*	?	?	?	?	?	?	?	?	?	?	?	?	?	?	?	?	?	?	?	?	?	?	?	?	?	0	?	?	?	?

	61	62	63	64	65	66	67	68	69	70	71	72	73	74	75	76	77	78	79	80	81	82	83	84	85	86	87	88	89	90
Rajiformes	0	0	0	1	1	2	1	1	0	0	0	0	1	1	1	1	1	1	2	0	0	0	1	1	0	1	0	1	0	1
Heterodontus	0	0	0	0	0	0	0	0	0	1	0	4	0	0	0	0	0	1	0	0	0	0	0	0	0	0	0	0	0	0
Orectolobiformes	0	0	0	0	0	0	0	0	0	1	0	4	0	0	0	0	0	1	0	0	0	0	0	0	0	0	1	0	0	2
Lamniformes	0	0	0	0	0	0	0	0	1	0	0	2	0	0	0	0	0	1	0	0	0	0	0	0	0	0	1	0	0	2
Carcharhiniformes	0	0	0	0	0	0	0	0	1	0	0	2	0	0	0	0	0	1	0	0	0	0	0	0	0	0	1	0	0	2
Chlamydoselachus	?	0	1	0	0	0	0	0	0	0	1	3	0	0	0	0	0	0	0	0	1	0	0	0	1	0	0	0	0	2
Hexanchus	1	2	0	0	0	0	0	0	0	0	1	3	1	0	0	1	0	0	0	0	1	1	0	0	1	0	0	0	1	1
Notorynchus	1	2	0	0	0	0	0	0	0	0	1	3	1	0	0	1	0	0	0	0	1	1	0	0	1	0	0	0	1	1
Heptranchias	1	2	0	0	0	0	0	0	0	0	1	3	1	0	0	1	0	0	0	0	1	1	0	0	1	0	0	0	1	1
Echinorhinus	0	0	1	0	0	0	0	0	0	0	1	3	1	0	0	1	0	1	0	3	0	0	0	0	0	0	0	0	0	1
Etmopteridae	0	1	0	0	0	0	0	0	0	0	0	1	1	0	0	1	0	1	0	0	0	0	0	0	0	0	0	0	0	0
Somniosidae	0	1	0	0	0	0	0	0	0	0	0	1	1	0	0	1	0	1	0	1	0	0	0	0	0	0	0	0	0	1
Oxynotus	0	1	0	0	0	0	0	0	0	0	0	1	1	0	0	1	0	1	0	1	0	0	0	0	0	0	0	0	0	0
Dalatiinae	0	1	0	0	0	0	0	0	0	0	0	1	1	0	0	1	0	1	0	4	0	0	0	0	0	0	0	0	0	1
Euprotomicrininae	0	1	0	0	0	0	0	0	0	0	0	1	1	0	0	1	0	1	0	3	0	0	0	0	0	0	0	0	0	1
Centrophoridae	0	1	0	0	0	0	0	0	0	0	0	1	1	0	0	1	0	1	0	2	0	0	0	0	0	0	0	0	0	0
Squalidae	0	1	0	0	0	1	0	1	0	0	0	0	1	1	1	1	1	1	0	0	0	0	0	0	0	0	0	0	0	0
Squatina	0	0	0	0	1	2	1	1	?	?	0	?	1	1	1	1	1	1	2	0	0	0	1	0	0	1	0	1	1	1
Pristiophoridae	0	1	1	1	1	2	1	1	0	0	0	0	1	1	1	1	1	1	2	0	0	0	1	1	0	0	0	0	0	1
†*Palaeocarcharias*	?	?	?	?	?	?	?	0	?	?	0	?	0	0	?	0	?	?	?	?	?	?	0	?	?	?	?	0	?	1

	91	92	93	94	95	96	97	98	99	100	101	102	103	104	105	106	107	108	109	110	111	112	113	114	115	116	117	118	119	120
Rajiformes	0	1	?	1	0	0	1	0	1	0	0	2	0	1	1	?	1	0	0	0	0	0	0	0	0	0	0	0	0	1
Heterodontus	?	?	0	0	0	0	0	0	0	1	0	2	0	0	0	0	0	0	0	0	0	0	0	1	1	0	1	0	0	1
Orectolobiformes	?	?	1	0	0	0	0	0	1	1	0	2	0	0	0	0	1	0	[01]	[01]	[01]	[01]	1	1	1	0	1	0	0	1
Lamniformes	?	?	1	0	0	0	0	0	1	1	0	[01]	1	1	1	0	1	0	0	0	0	0	0	0	0	0	1	0	0	1
Carcharhiniformes	?	?	1	0	0	0	0	0	1	1	0	0	0	[01]	[01]	0	1	[01]	[01]	[01]	[01]	[01]	1	1	0	1	0	0	1	1
Chlamydoselachus	?	0	1	0	1	0	0	0	0	0	0	0	0	0	0	0	1	1	1	1	0	1	0	1	1	1	1	0	0	1
Hexanchus	1	0	0	0	1	0	0	0	0	0	1	0	1	0	1	1	1	1	0	0	0	0	0	1	1	1	1	0	0	0
Notorynchus	1	0	0	0	1	1	0	0	0	0	1	0	1	0	1	1	1	1	0	0	0	0	0	1	1	1	1	0	0	0
Heptranchias	1	0	0	0	1	1	0	0	0	0	1	0	0	0	1	1	1	1	1	0	0	1	0	1	1	1	1	0	0	0
Echinorhinus	0	0	?	1	0	0	0	0	1	0	0	0	0	0	0	0	1	0	1	0	0	0	0	1	1	0	1	0	0	0

Etmopteridae	0	0	?	1	0	0	0	0	1	0	0	2	0	0	0	0	0	0	1	0	1	0	0	1	1	1	1	0	1	0
Somniosidae	0	0	?	1	0	0	0	1	1	0	0	2	0	0	0	0	[01]	0	1	0	0	0	0	1	1	1	1	0	1	0
Oxynotus	0	0	?	1	0	0	0	1	1	0	0	2	0	0	0	0	0	0	0	0	0	0	0	1	1	1	1	0	0	0
Dalatiinae	0	0	?	1	0	0	0	1	1	0	0	2	0	0	0	0	1	0	1	0	1	0	0	1	1	1	1	0	1	0
Euprotomicrininae	2	0	?	1	0	0	0	1	1	0	0	2	0	0	0	0	1	0	1	0	1	0	0	1	1	1	1	0	1	0
Centrophoridae	0	0	?	1	0	0	0	0	1	0	0	2	0	0	0	0	0	0	0	0	0	0	0	1	1	1	1	0	1	0
Squalidae	0	0	?	1	0	0	0	0	1	0	0	2	0	0	0	0	0	0	0	0	0	0	0	1	0	1	1	0	0	0
Squatina	0	1	?	1	0	0	1	0	1	0	0	2	0	0	0	0	1	0	0	0	1	0	1	1	1	0	1	1	0	1
Pristiophoridae	0	1	?	1	0	0	1	0	1	0	0	2	?	?	?	?	1	0	0	0	1	0	1	1	1	0	1	1	0	1
†*Palaeocarcharias*	?	0	1	0	0	0	?	0	?	?	?	1	?	?	?	?	1	?	0	1	0	0	1	1	1	0	1	1	0	1

	**121**	**122**	**123**	**124**	**125**	**126**	**127**	**128**	**129**	**130**	**131**	**132**	**133**	**134**	**135**	**136**
Rajiformes	0	1	0	1	1	2	3	3	0	0	1	0	0	1	0	0
Heterodontus	1	0	0	0	0	2	0	0	0	0	0	0	0	1	0	0
Orectolobiformes	1	0	0	0	0	2	1	0	0	0	0	1	0	1	0	0
Lamniformes	0	1	[01]	0	1	2	1	0	0	0	1	0	1	0	0	0
Carcharhiniformes	0	1	0	0	1	2	2	0	0	0	0	0	1	1	0	0
Chlamydoselachus	1	0	0	0	0	1	0	1	0	0	0	0	1	1	0	1
Hexanchus	0	0	0	0	0	0	0	2	1	0	0	0	1	1	1	2
Notorynchus	0	0	0	0	0	0	0	2	1	0	0	0	1	1	1	2
Heptranchias	0	0	0	0	0	0	0	2	1	0	0	0	1	1	1	2
Echinorhinus	0	0	0	0	0	0	0	0	1	1	0	0	1	1	1	2
Etmopteridae	0	0	0	0	0	2	0	0	2	0	0	0	?	?	?	?
Somniosidae	0	0	0	0	0	2	0	0	2	0	0	0	?	?	?	?
Oxynotus	0	0	0	0	0	2	0	0	2	0	0	0	?	?	?	?
Dalatiinae	0	0	0	0	0	2	0	0	2	0	0	0	?	?	?	?
Euprotomicrininae	0	0	0	0	0	2	0	0	2	0	0	0	?	?	?	?
Centrophoridae	0	0	0	0	0	2	0	0	2	1	0	0	?	?	?	?
Squalidae	0	0	0	0	0	2	0	0	2	2	1	0	?	?	?	?
Squatina	1	0	1	0	0	2	0	0	0	0	1	0	0	1	0	0
Pristiophoridae	1	0	1	1	0	2	0	0	0	0	1	0	0	1	1	0
†*Palaeocarcharias*	0	1	1	?	0	?	?	0	1	1	?	1	1	0	0	0

Supplementary Literature:

Applegate, S.P. The origin of the lamniform sharks, a study in morphology and paleontology of recent and fossil genera. American Elasmobranch Society, Annual meeting, Abstract, p. 1. (2001).

Bernárdez, E. *Truyolsodontos estauni* n. gen., n. sp., Truyolsodontidae, a new family of lamniform sharks from the Cenomanian of northern Spain. *Ann. Paleontol.* **104,** 175-181 (2018).

Cappetta, H. *Handbook of Paleoichthyology, Vol 3B: Chondrichthyes II*. Stuttgart. 1-192 (Gustav Fischer Verlag, 1987).

Cappetta, H. *Handbook of Paleoichthyology, Vol 3E: Chondrichthyes - Mesozoic and Cenozoic Elasmobranchii: Teeth.* München. 1-512 (Verlag Dr. Friedrich Pfeil, 2012).

de Beaumont, G. Observations préliminaires sur trois Sélaciens nouveaux du calcaire lithographique d'Eichstätt (Bavière). *Eclogae Geol. Helv.* **53,** 315-328 (1960).

Duffin, C. J. The upper jurassic selachian *Palaeocarcharias* de Beaumont (1960). *Zool. J. Linn. Soc.* **94,** 271-286 (1988).

Goloboff, P. A., Farris, J. S. & Nixon, K. C. 2008. TNT, a free program for phylogenetic analysis. Cladistics, 24, 774–786.

Goto, M. Histological structure of the teeth, dermal and mucous denticles and gill rakers of a female megamouth shark, *Megachasma pelagious*, from Hakata Bay, Japan. *ACBTE*. **6,** 9-18 (1999).

Hoffman, B. L., Hageman, S. A., Claycomb, G. D. Scanning electron microscope examination of the dental enameloid of the Cretaceous durophagous shark *Ptychodus* supports neoselachian classification. *J. Paleontol.* **90,** 741-762 (2016).

Jambura, P. L., Pfaff, C., Underwood, C. J., Ward, D. J., Kriwet, J. Tooth mineralization and histology patterns in extinct and extant snaggletooth sharks, *Hemipristis* (Carcharhiniformes, Hemigaleidae)-Evolutionary significance or ecological adaptation? *PloS ONE*. **13,** e0200951; 10.1371/journal.pone.0200951 (2018).

Kakizawa, Y. On the teeth of salmon shark, *Lamna ditropis* Hubbs & Follet. *Nihon Univ. dent. J.* **58,** 59-69 (1984).

Klug, S. Monophyly, phylogeny and systematic position of the †Synechodontiformes (Chondrichthyes, Neoselachii). Zool. Scr. 39, 37-49 (2010).

Landemaine, O., Thies, D., Waschkewitz, J. The Late Jurassic shark *Palaeocarcharias* (Elasmobranchii, Selachimorpha) – functional morphology of teeth, dermal cephalic lobes and phylogenetic position. *Palaeontogr. Abt. A Palaeozool-Stratigr.* **312,** 103-165 (2018).

Maddison, W. P. & Maddison, D. R. (2008). Mesquite: a modular system for evolutionary analysis. Version 3.03. Updated at: http://mesquiteproject.org.

Møller, I.J., Melsen, B., Jensen, S.J., Kirkegaard E. A histological, chemical and x-ray diffraction study on contemporary (Carcharias glaucus) and fossilized (Macrota odontaspis) shark teeth. *Archs*. *oral Biol*. **20,** 797-802 (1975).

Moyer, J. K., Riccio, M. L., Bemis, W. E. Development and microstructure of tooth histotypes in the blue shark, *Prionace glauca* (Carcharhiniformes: Carcharhinidae) and the great white shark, *Carcharodon carcharias* (Lamniformes: Lamnidae). *J. Morphol*. **276,** 797-817 (2015a).

Moyer, J. K., Hamilton, N. D., Hadlock Seeley, R., Riccio, M. L., Bemis, W. E. Identification of shark teeth (Elasmobranchii: Lamnidae) from a historic fishing station on Smuttynose Island, Maine, using computed tomography imaging. *Northeast. Nat.* **22,** 585-597 (2015b).

Nixon, K.C. 2002. Winclada, v. 1.00.08. Program and documentation available at www.cladistics.com

Schnetz, L., Pfaff, C., Kriwet, J. Tooth development and histology patterns in lamniform sharks (Elasmobranchii, Lamniformes) revisited. *J. Morphol*. **277,** 1584–1598 (2016).

Shirai, S. Phylogenetic interrelationships of Neoselachians (Chondrichthyes: Euselachii). In: Staiassny, M.L.J., Parenti, L.R., Johnson, G.D. (eds): Interrelationships of fishes. Academic Press 9-34 (1996).

Yabumoto, Y., Goto, M., Yano, K., Uyeno, T. Dentition of a female megamouth, *Megachasma pelagios*, collected from Hakata Bay, Japan in *Biology of the Megamouth Shark* (eds: Yano, K., Morrissey, J., F., Yabumoto, Y., Nakaya, K.) 63-75 (Tokai University Press, 1997).
